# Supplementary material for: Poldip2 promotes mtDNA elimination during Drosophila spermatogenesis to ensure maternal inheritance
Source: EMBO J. 2025 Feb 11;44(6):1724–48. doi: 10.1038/s44318-025-00378-4 (PMC11914606; doi:10.1038/s44318-025-00378-4)
Supplement: Supplementary file 1 — Appendix [file 44318_2025_378_MOESM1_ESM.pdf]

Poldip2 promotes mtDNA elimination during *Drosophila* spermatogenesis to ensure maternal inheritance

Ziming Wang[1,2†], Tirawit Meerod[3†], Nuria Cortes-Silva[1,2,4†], Ason C-Y Chiang[3†], Ziyan Nie[1,2,5], Ying Di[1,2,6], Peiqiang Mu[7], Ankit Verma[1], Adam James Reid[1], Hansong Ma[3*]

Appendix

Table of Content

Appendix Table S1. *EMS-23* mutations and mapping results 2

Appendix Table S2: Primers used in this study 3

Appendix Table S1: *EMS-23* mutations and mapping results

Gene	Mutation type	NT change	Impact	Df cross	mtDNA retention
pcyt1	missense	c.308C>T	moderate	Df(3L)BSC289	wildtype
pxn	missense	c.1729G>A	moderate	Df(3L)BSC119	wildtype
		c.3790G>A		Df(3L)BSC23	wildtype
Ir64a	missense	c.434G>A	moderate	Df(3L)BSC371	wildtype
		c.1822G>A		Df(3L)Exel6105	wildtype
CG7504	frameshift	c.1131delA	high	Df(3L)Exel6112	wildtype
				Df(3L)BSC388	wildtype
CG5653	missense	c.568G>A	moderate	Df(3L)ED4421	wildtype
tna	missense	c.2839C>T	moderate	Df(3L)ED4457	wildtype
CG6793	stop_gained	c.109C>T	high	Df(3L)ED4475	wildtype
CG32104	missense	c.1684G>A	moderate	Df(3L)ED4475	wildtype
CG14115	missense	c.604G>A	moderate	Df(3L)BSC12	wildtype
bbg	missense	c.2585G>A	moderate	Df(3L)ED4543	wildtype
CTPsyn	missense	c.74C>T	moderate	Df(3L)ED217	wildtype
rpn1	missense	c.2701C>T	moderate	Df(3L)ED4858	wildtype
CG5664	missense	c.1942A>T	moderate	Df(3L)BSC419	wildtype
orco	missense	c.523G>A	moderate	Df(3R)ED5156	wildtype
poldip2	missense	c.895C>T	moderate	BDSC17500	Medium/high
osi17	missense	c.670C>T	moderate	Df(3R)BSC681	wildtype
				Df(3R)BSC745	wildtype
fst	conservative_inframe_insertion	c.346_357dupCCACCACCACCA	moderate	Df(3R)ED5428	wildtype
				Df(3R)Exel6264	wildtype
CG42727	frameshift	c.558delC	high	Df(3R)ED5705	wildtype
lkb1	missense	c.1190C>T	moderate	Df(3R)ED5623	wildtype
sxe2	missense	c.535C>T	moderate	Df(3R)Exel7328	wildtype
cstF64	missense	c.404C>T	moderate	Df(3R)ED2	wildtype
CG4662	missense	c.1335G>T	moderate	Df(3R)ED6025	wildtype
wda	missense	c.761G>A	moderate	Df(3R)Exel6280	wildtype
				Df(3R)ED6103	wildtype
CG12877	missense	c.2882T>A	moderate	DF(3R)BSC497	wildtype
		c.2887C>A			
NSD	missense	c.376G>A	moderate	Df(3R)ED6280	wildtype
apc	missense	c.509G>A	moderate	Df(3R)BSC874	wildtype
tmod	missense	c.1313C>T	moderate	Df(3R)BSC504	wildtype
		c.1333C>G		Df(3R)Exel7378	wildtype
		c.1339G>A			

Appendix Table S2: Primers used in this study

Primer	Sequence
mt:yak/mel common 6237F, ddPCR	CTTTTAATGGTTAAATTCCATTTATA
mt:yak/mel common 6314R, ddPCR	TTATTATTACAATGAAAATGTAAGGT
mt:mel specific 1847F, ddPCR	TTTATCCACCTTTATCCGCTGGAATT
mt:mel specific 2076R, ddPCR	TGCTAGTACTGGAAGTGATAATAATAA
HEXA F	TGTCAAGTGCTTCGTAACGTATGTG
HEXA R	GTGCTGCGGTATGGCATAGATG
mt361F	CTTTTATCCCCCTATTAAGAG
mt409R	GAAGCTTCTGTAGATATTAAATTATTA
mt2126F	TTGACCCAGCGGGAGGAGGAGAT
mt2194R	CTTCAGGGTGACCAAAAAATC
mt4357F	CATTTAACTTTAACTTTATCTTTAGCT
mt4444R	GCAAATATATGTTGTGTATGATTAATTC
mt6570F	CAACTAATTGA CCATAATTTAAAGG
mt6636R	TGATTTATACCTTATATTAGAACTTATGG
mt8701F	TATGAGCAACAGATGAATAAGC
mt8750R	ACGTCAAACTGATTTAAAGGC
mt10884F	GTAATAGGAACAGCTTTTATAGG
mt10938R	AAAATGATATTTGTCCTCAAGG
mt13245F	CGTCCAACCATTCATTCCAGCC
mt13321R	GGCCGCAGTATTTTGACTGTG
