# Supplementary figures and images for: Poldip2 promotes mtDNA elimination during Drosophila spermatogenesis to ensure maternal inheritance
Source: EMBO J. 2025 Feb 11;44(6):1724–48. doi: 10.1038/s44318-025-00378-4 (PMC11914606; doi:10.1038/s44318-025-00378-4)

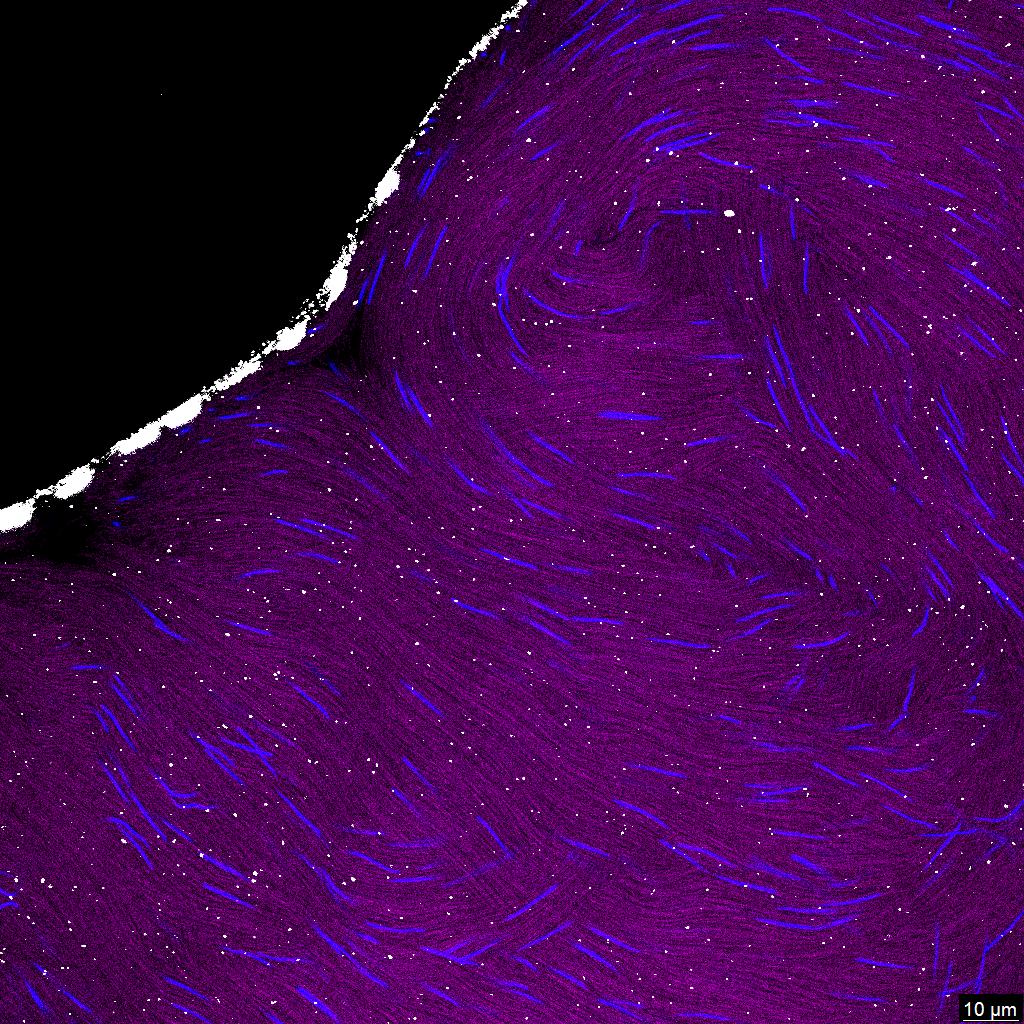

Supplement: Supplementary file 3 — Source data Fig. 1 [file 44318_2025_378_MOESM3_ESM.zip › Figure 1/1C/EMS-717.tif]

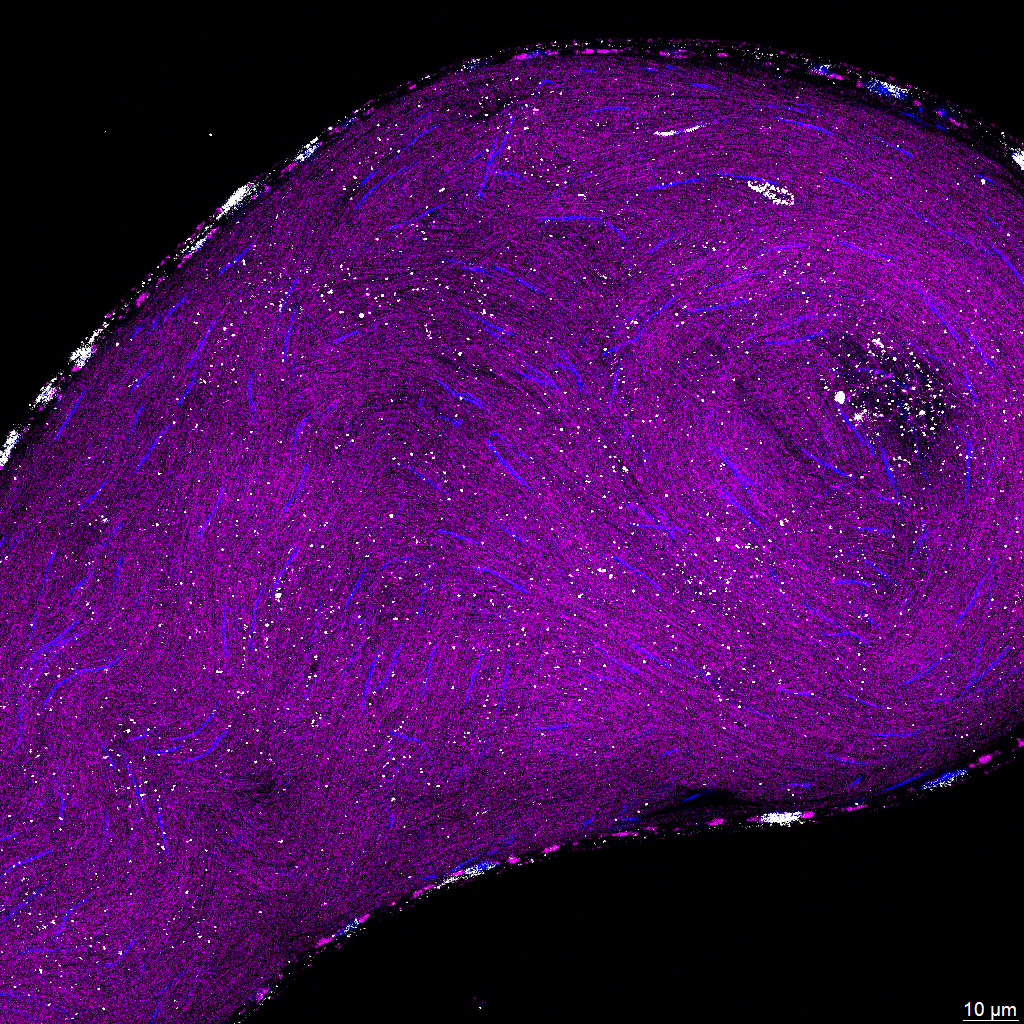

Supplement: Supplementary file 3 — Source data Fig. 1 [file 44318_2025_378_MOESM3_ESM.zip › Figure 1/1C/EMS-1094.tif]

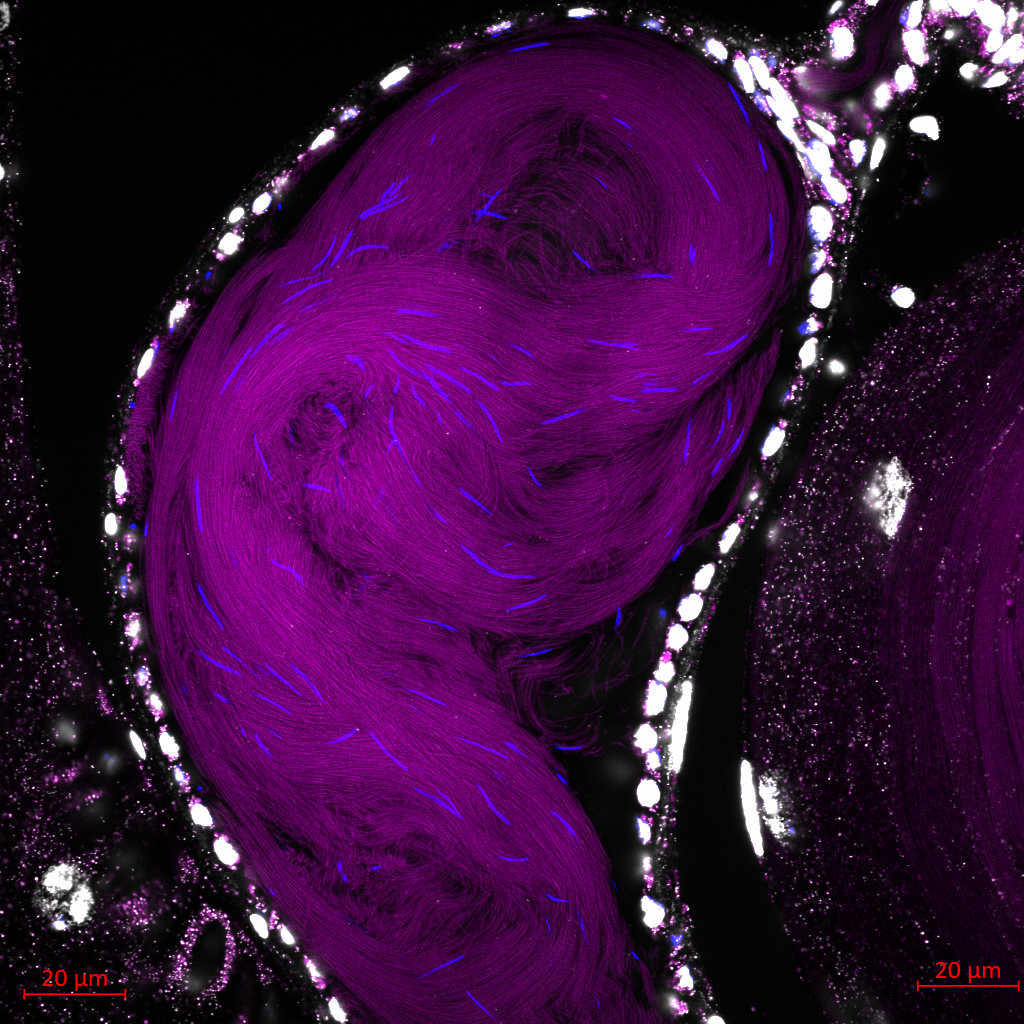

Supplement: Supplementary file 3 — Source data Fig. 1 [file 44318_2025_378_MOESM3_ESM.zip › Figure 1/1C/WT.tif]

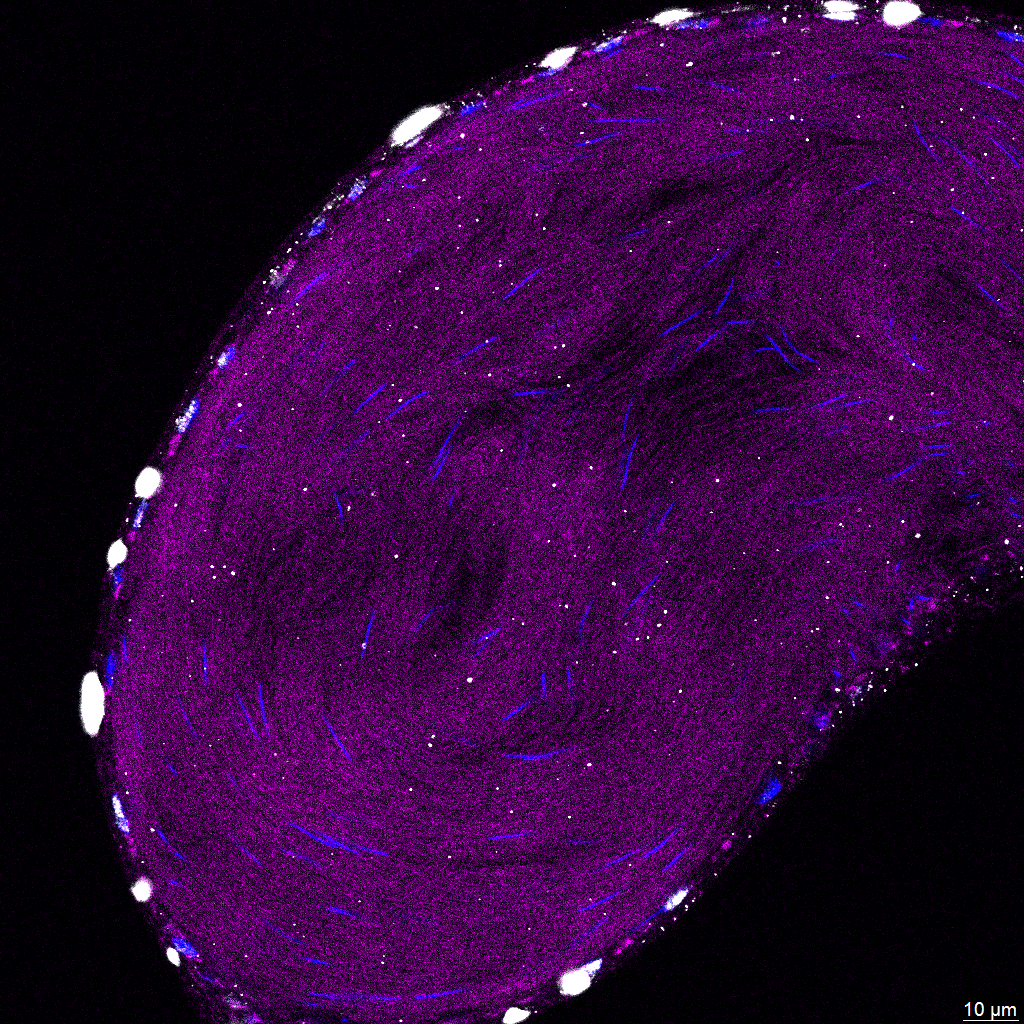

Supplement: Supplementary file 3 — Source data Fig. 1 [file 44318_2025_378_MOESM3_ESM.zip › Figure 1/1C/EMS-423.tif]

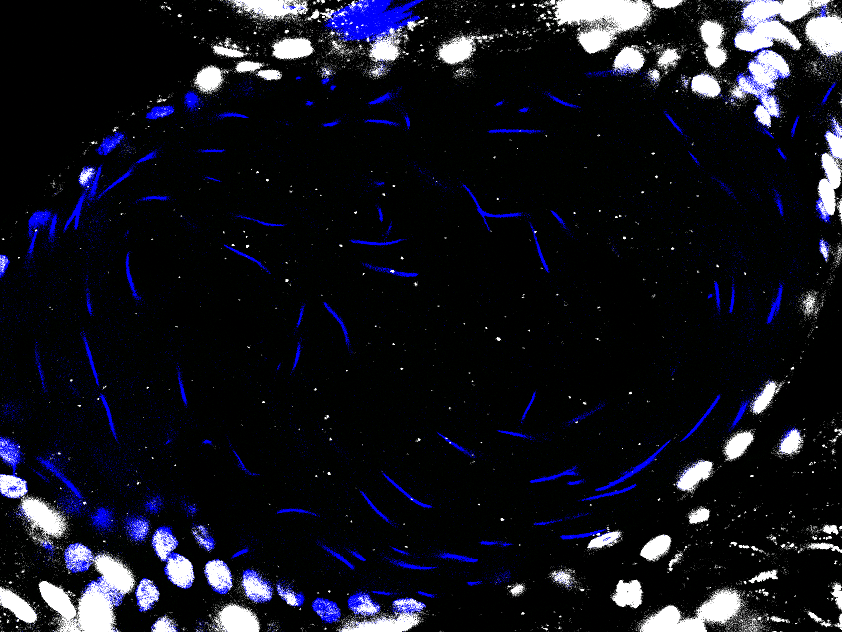

Supplement: Supplementary file 4 — Source data Fig. 2 [file 44318_2025_378_MOESM4_ESM.zip › Figure 2/2A/EMS23.tif]

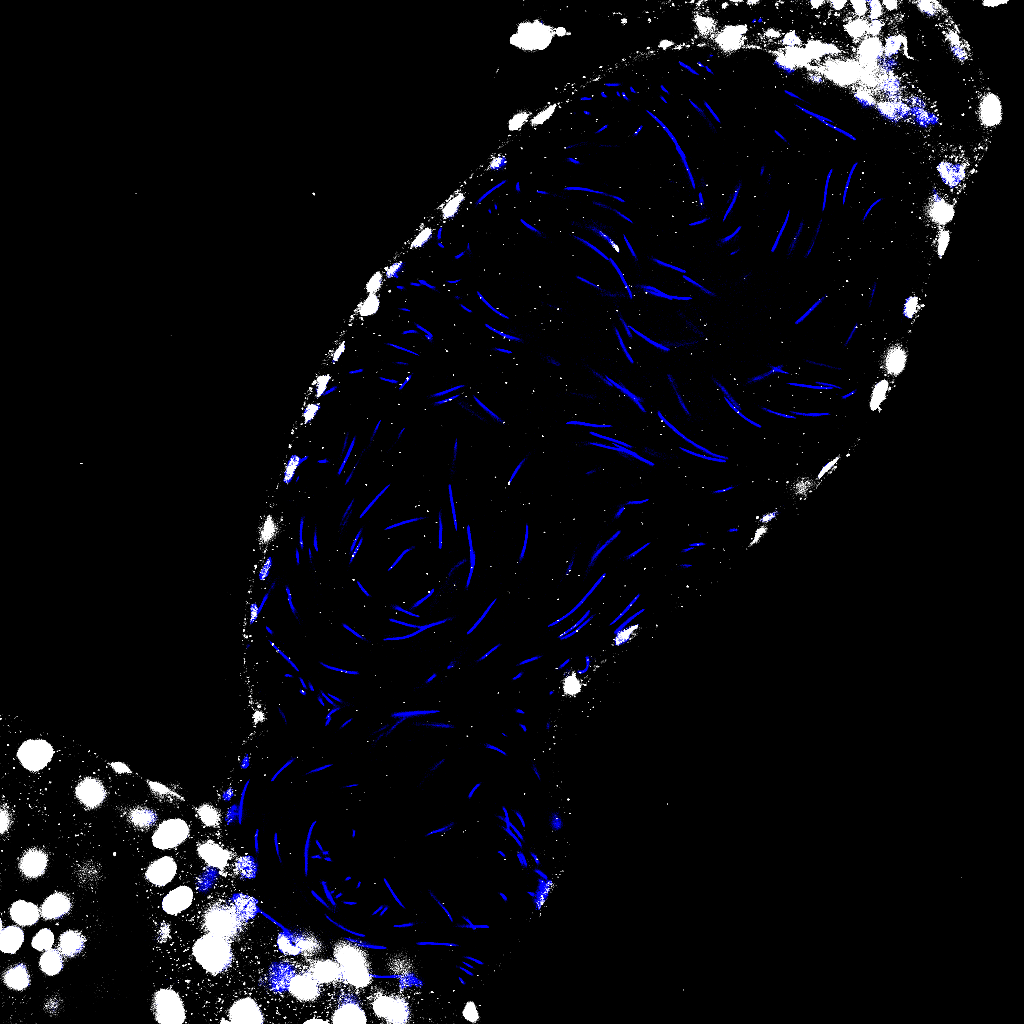

Supplement: Supplementary file 4 — Source data Fig. 2 [file 44318_2025_378_MOESM4_ESM.zip › Figure 2/2A/EMS23:Poldip2 EY08866.tif]

Fig 2C

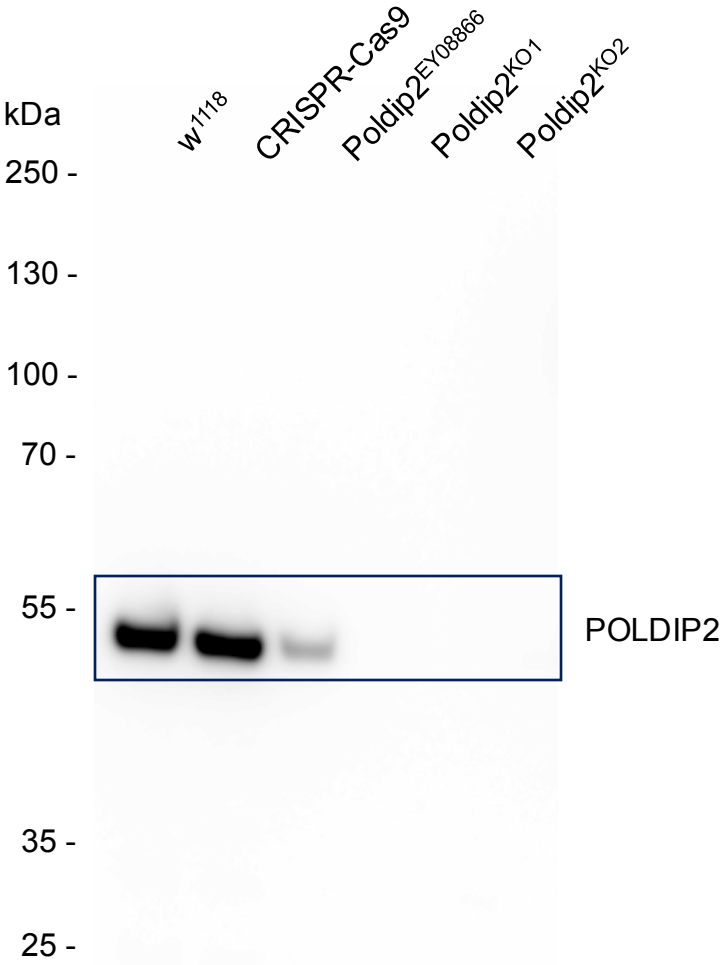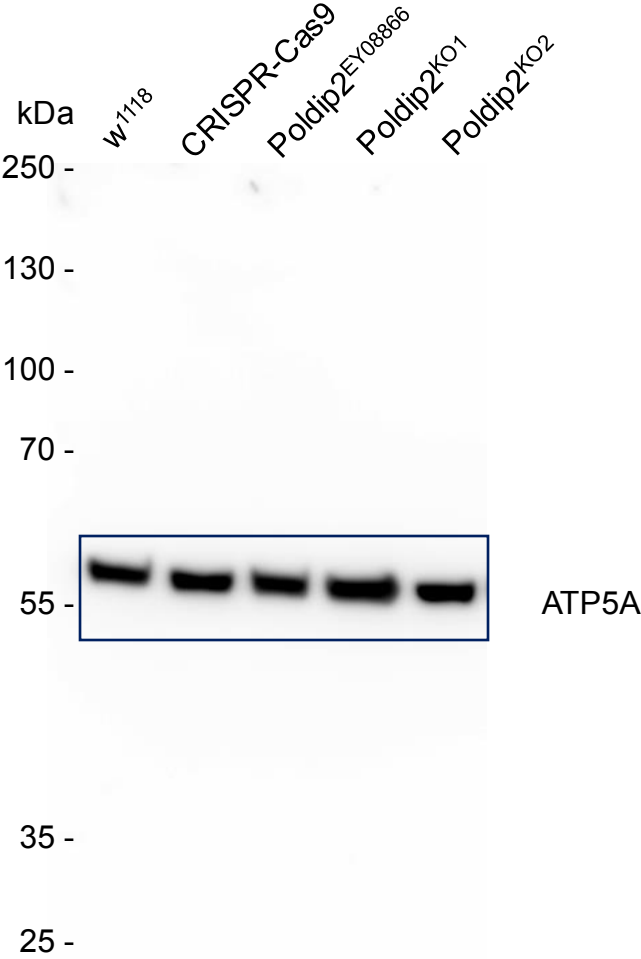

Supplement: Supplementary file 4 — Source data Fig. 2 [file 44318_2025_378_MOESM4_ESM.zip › Figure 2/2C/Poldip2 mutant blots.pdf]

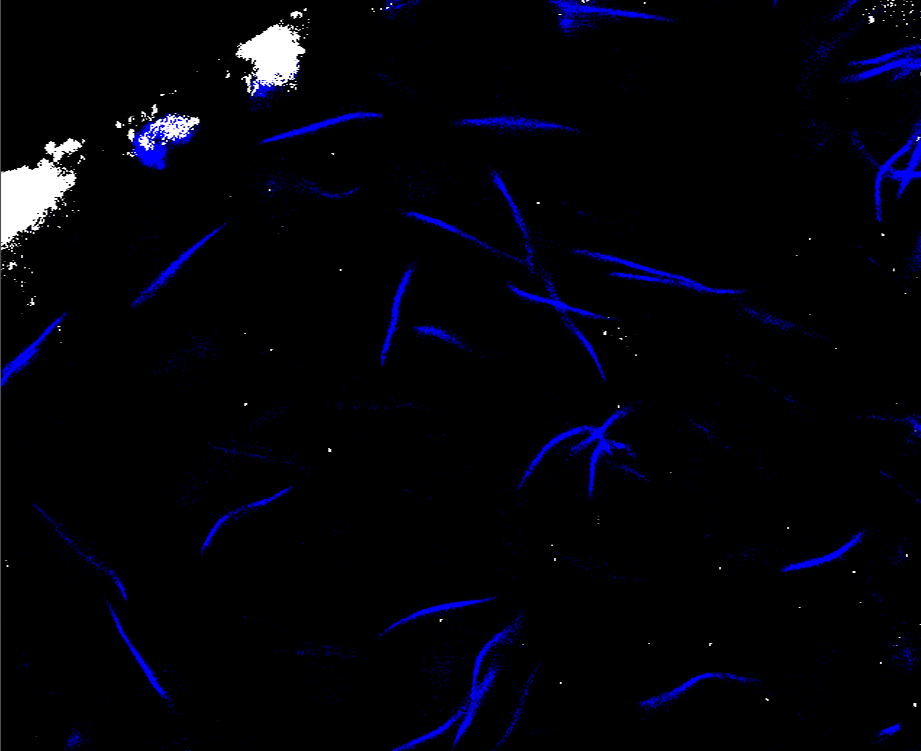

Supplement: Supplementary file 4 — Source data Fig. 2 [file 44318_2025_378_MOESM4_ESM.zip › Figure 2/2D/ubi-Poldip2 & poldip2 mutant.tif]

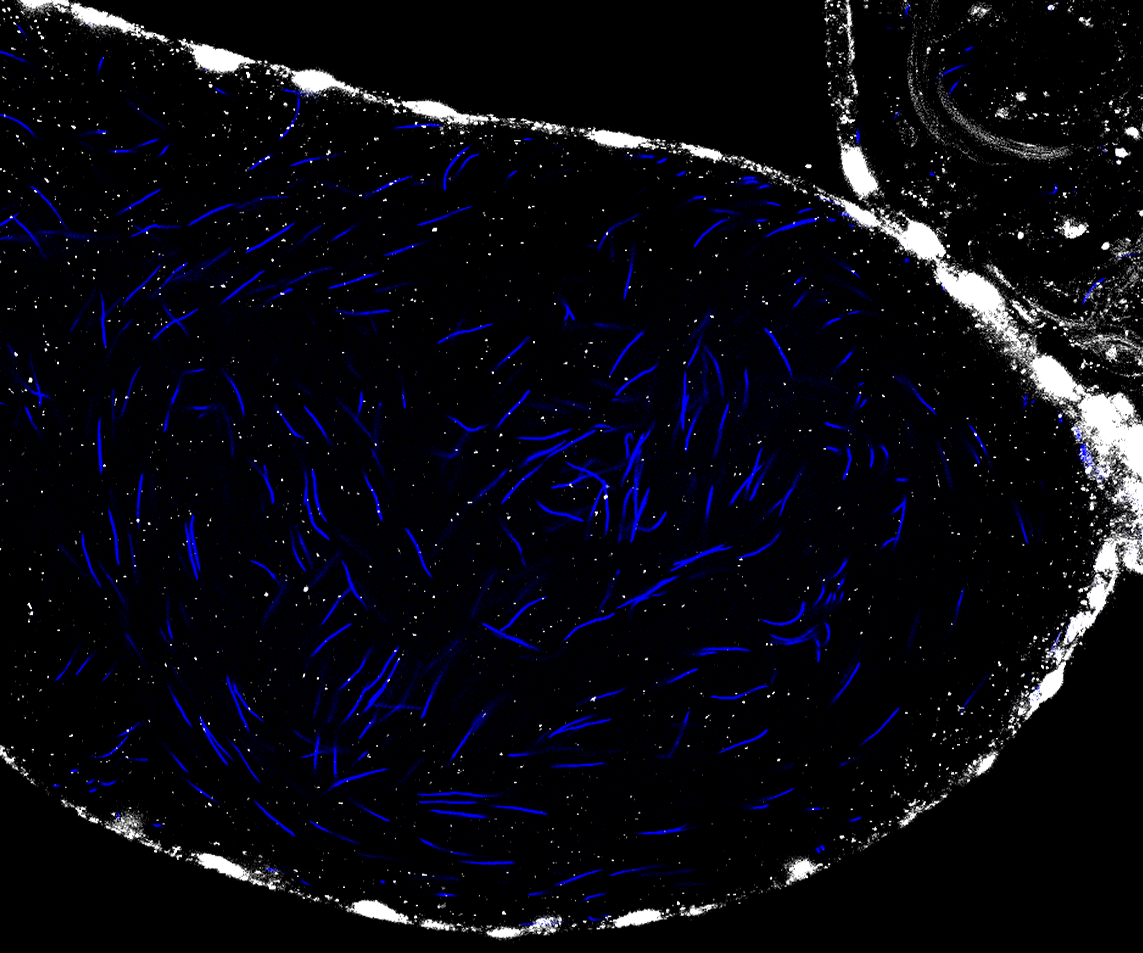

Supplement: Supplementary file 4 — Source data Fig. 2 [file 44318_2025_378_MOESM4_ESM.zip › Figure 2/2D/POLDIP2 mutant.tif]

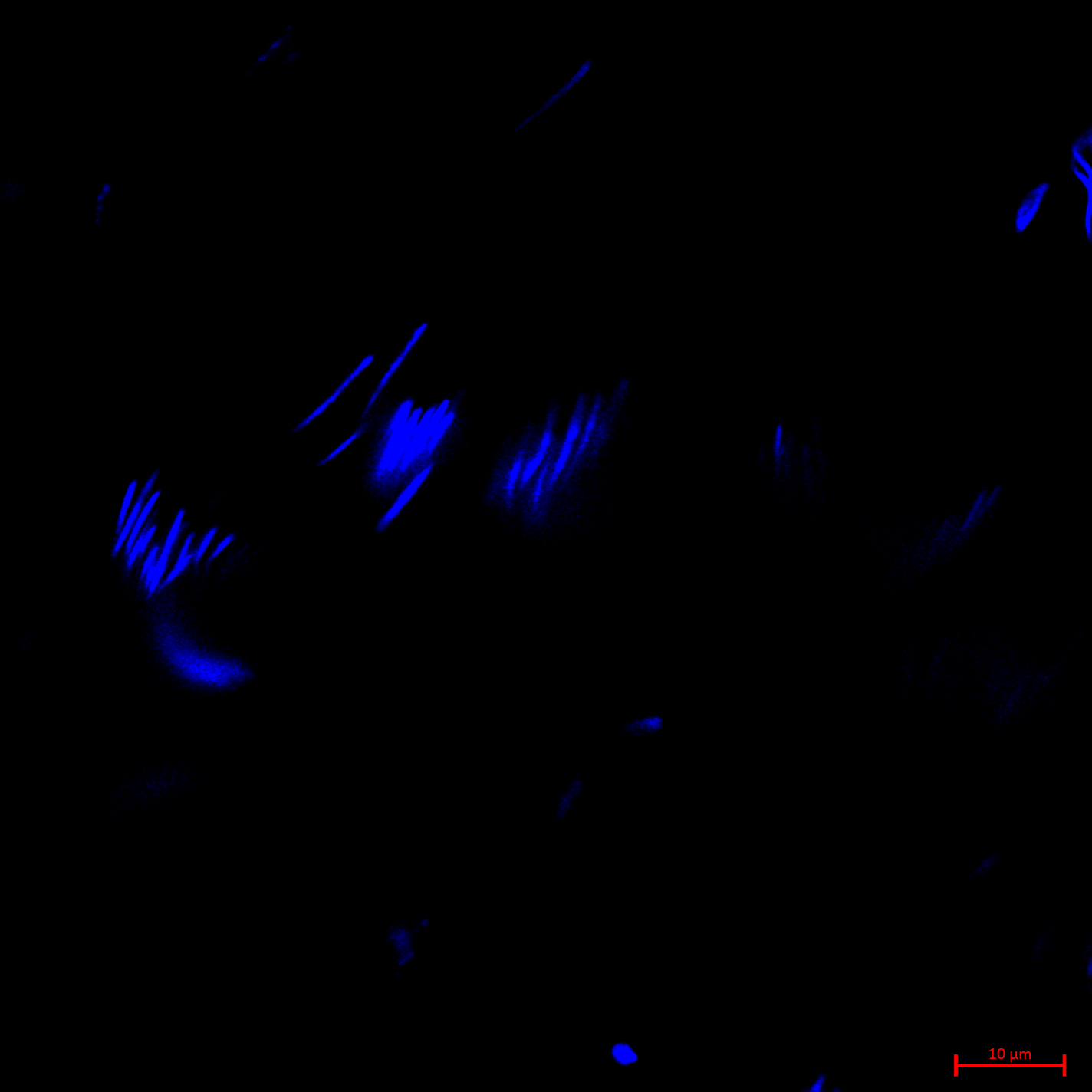

Supplement: Supplementary file 5 — Source data Fig. 3 [file 44318_2025_378_MOESM5_ESM.zip › Figure 3/3C/PA-mCherry DJ-GFP 1.tif]

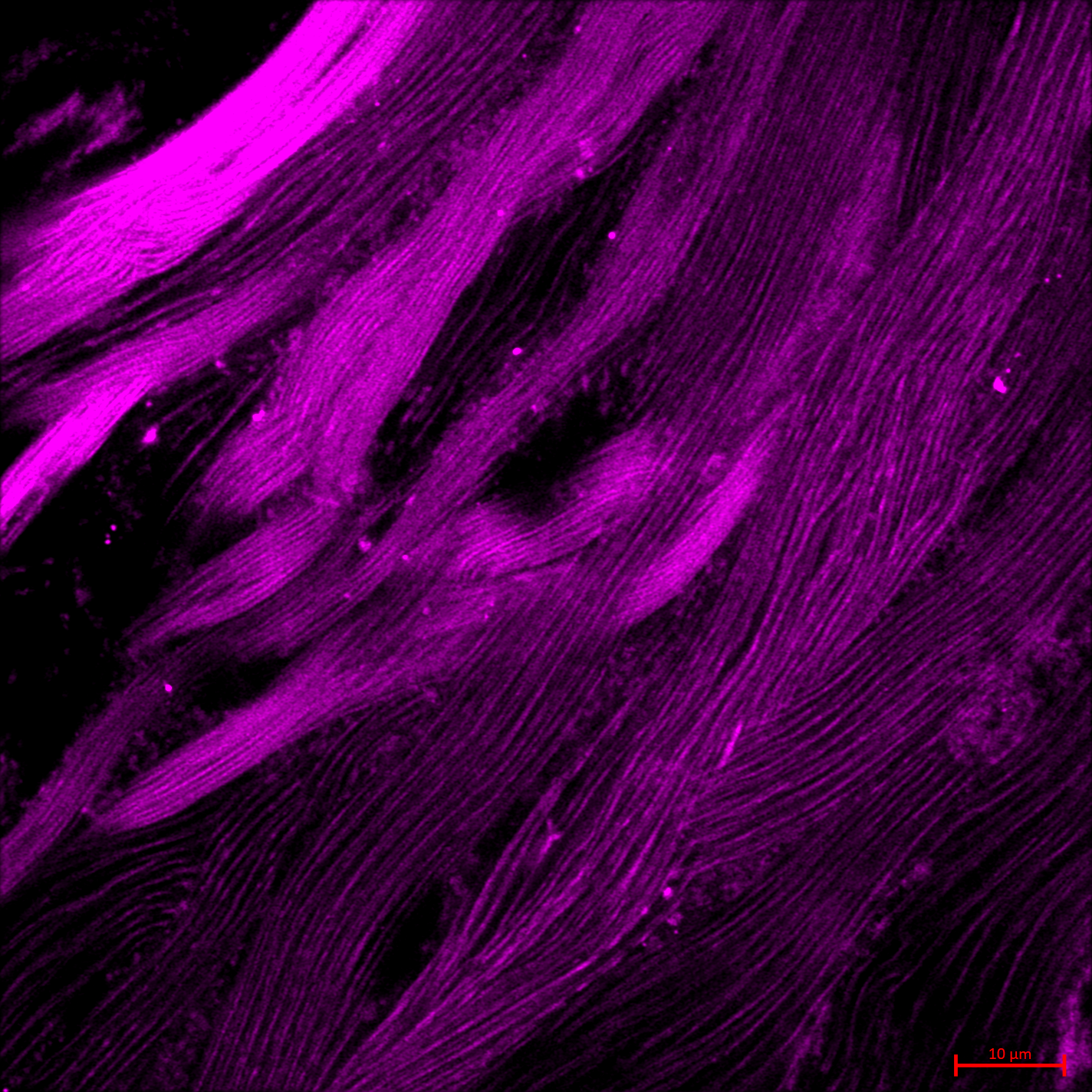

Supplement: Supplementary file 5 — Source data Fig. 3 [file 44318_2025_378_MOESM5_ESM.zip › Figure 3/3C/PA-mCherry DJ-GFP 3.tif]

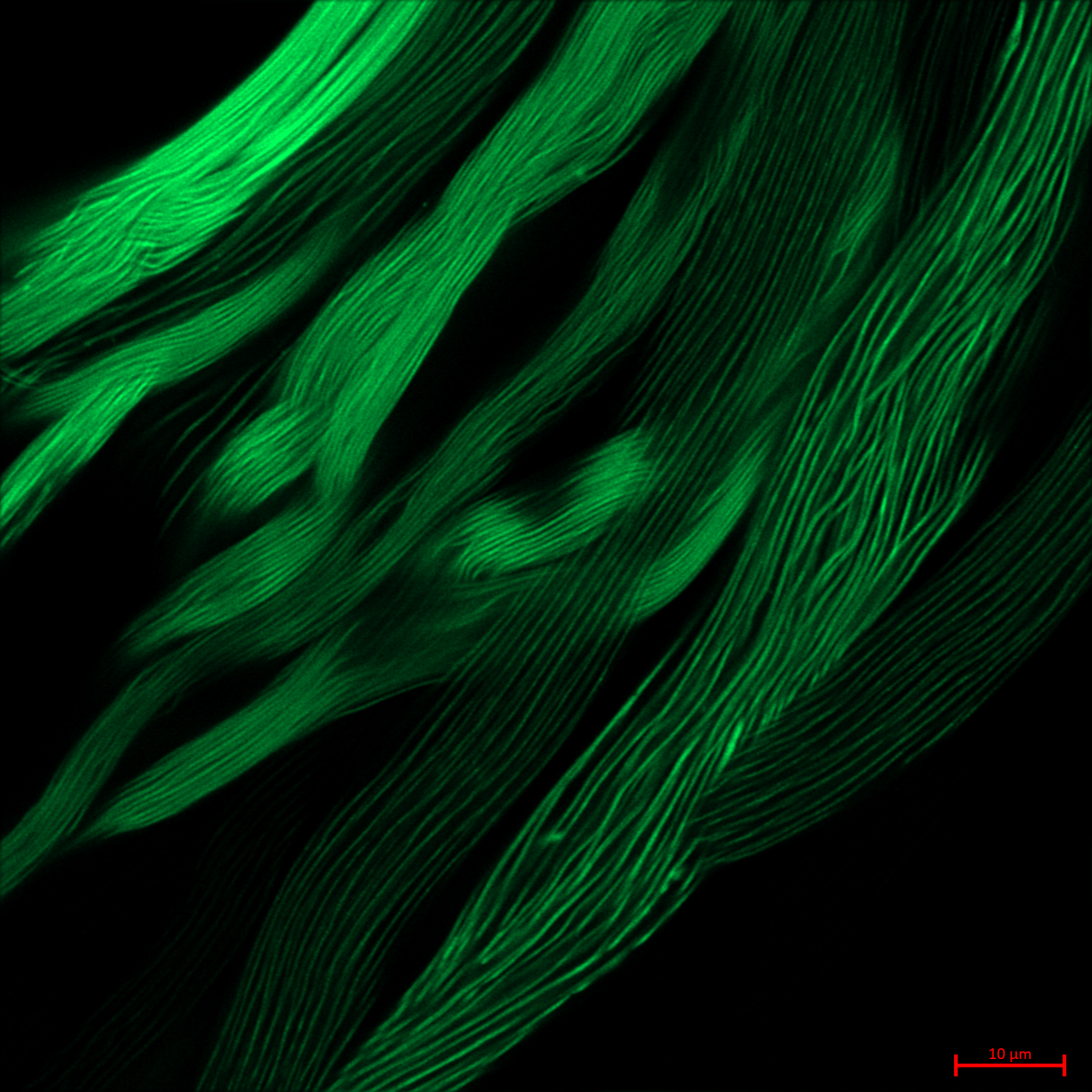

Supplement: Supplementary file 5 — Source data Fig. 3 [file 44318_2025_378_MOESM5_ESM.zip › Figure 3/3C/PA-mCherry DJ-GFP 2.tif]

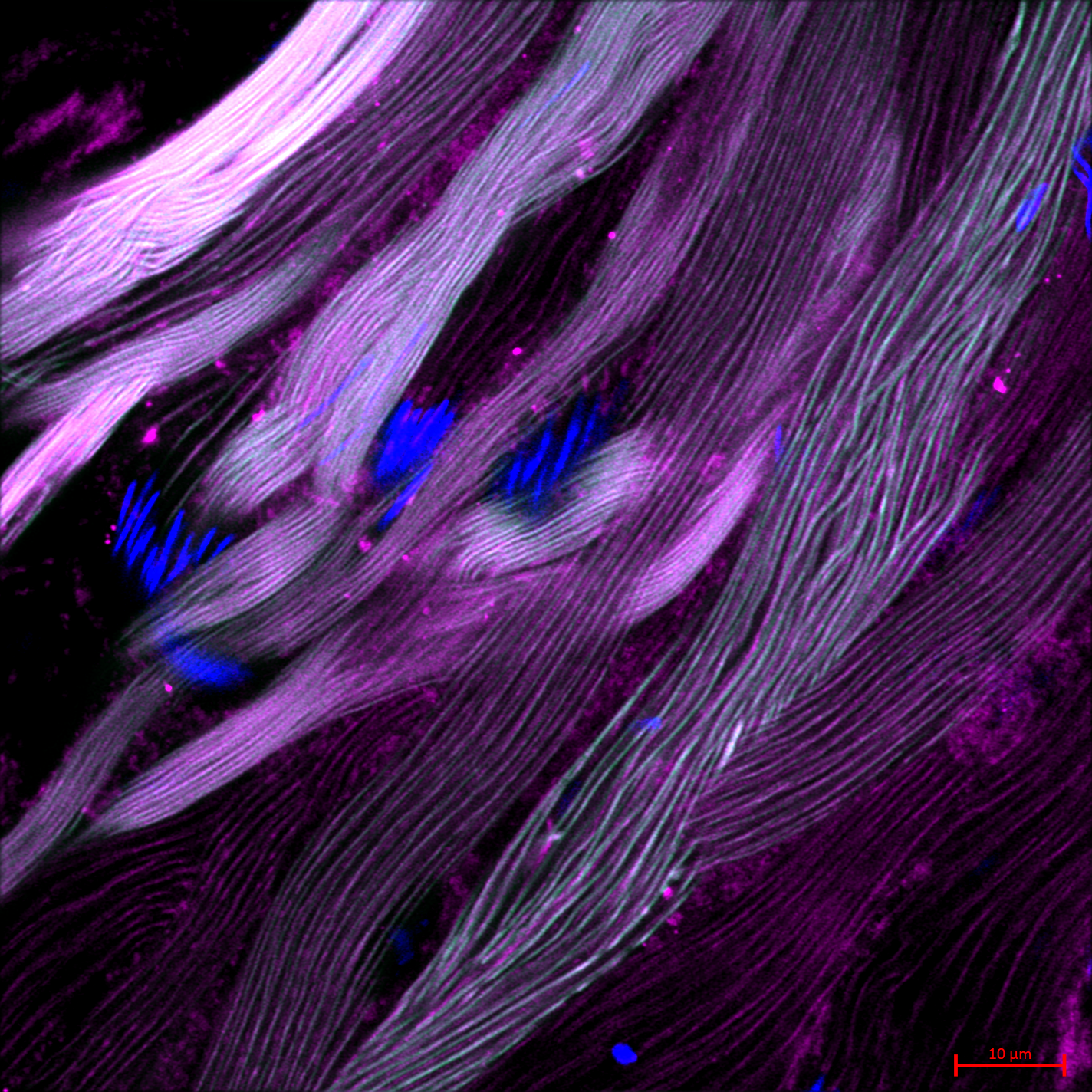

Supplement: Supplementary file 5 — Source data Fig. 3 [file 44318_2025_378_MOESM5_ESM.zip › Figure 3/3C/PA-mCherry DJ-GFP 4.tif]

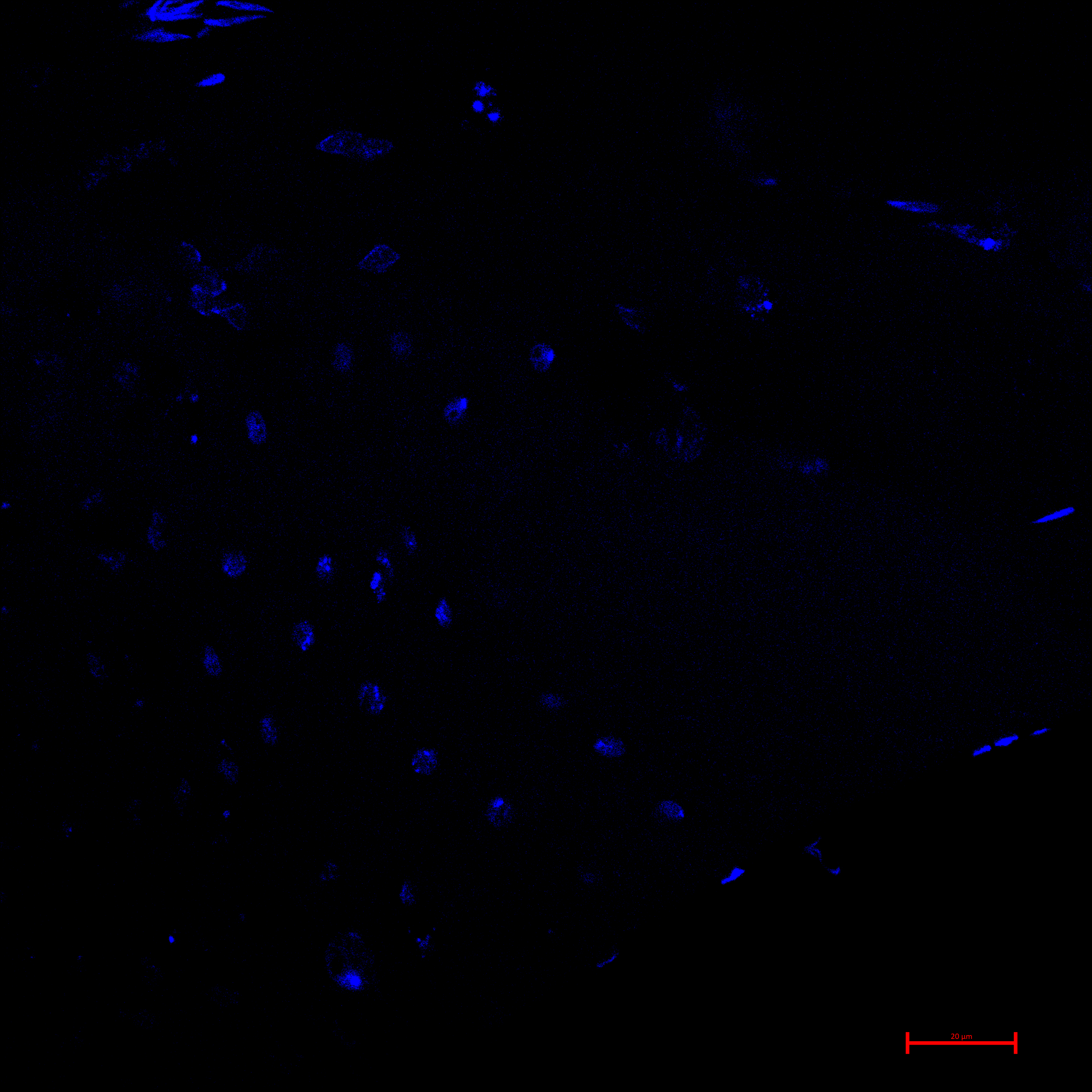

Supplement: Supplementary file 5 — Source data Fig. 3 [file 44318_2025_378_MOESM5_ESM.zip › Figure 3/3C/PB-mCherry tom20 GFP 1.tif]

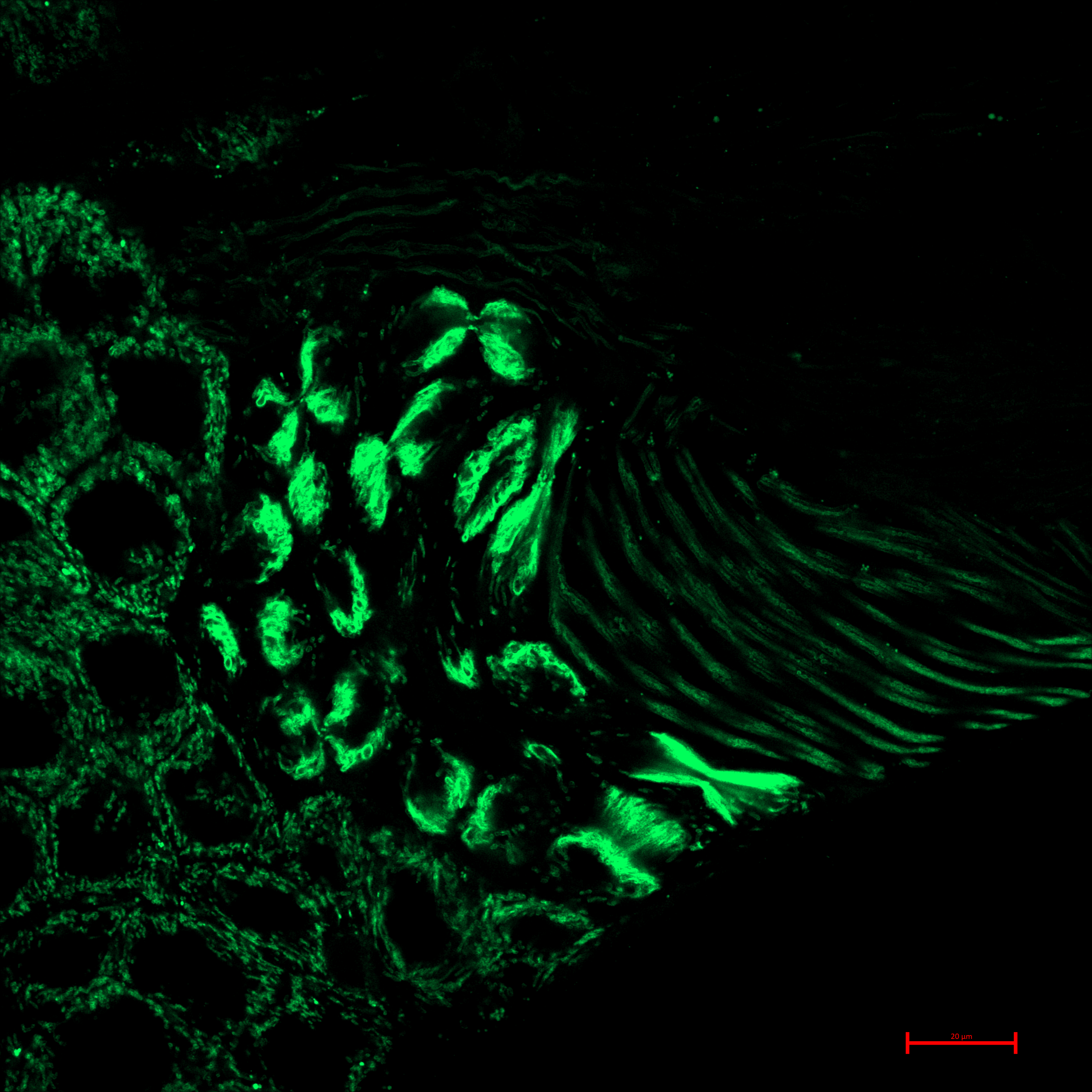

Supplement: Supplementary file 5 — Source data Fig. 3 [file 44318_2025_378_MOESM5_ESM.zip › Figure 3/3C/PB-mCherry tom20 GFP 2.tif]

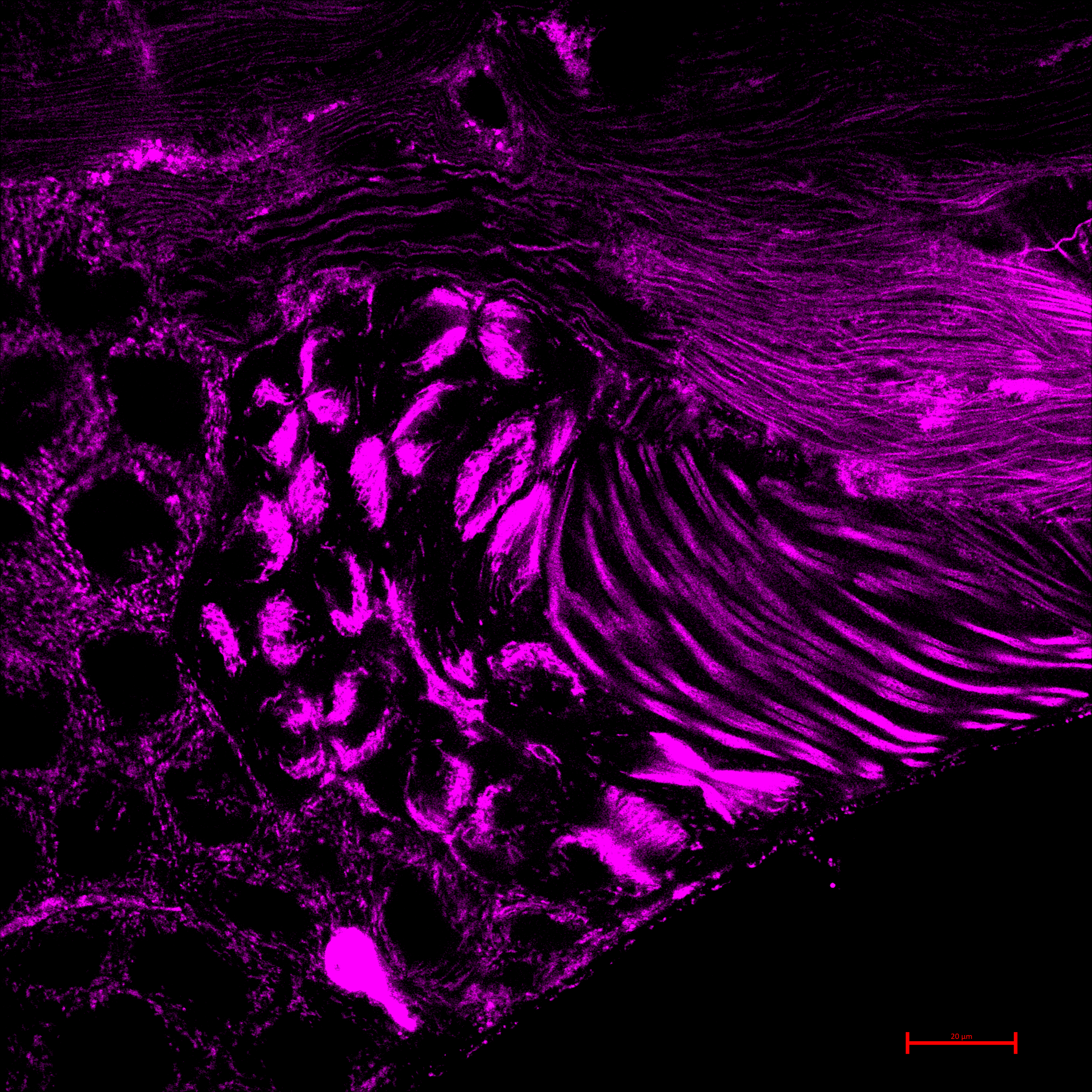

Supplement: Supplementary file 5 — Source data Fig. 3 [file 44318_2025_378_MOESM5_ESM.zip › Figure 3/3C/PB-mCherry tom20 GFP 3.tif]

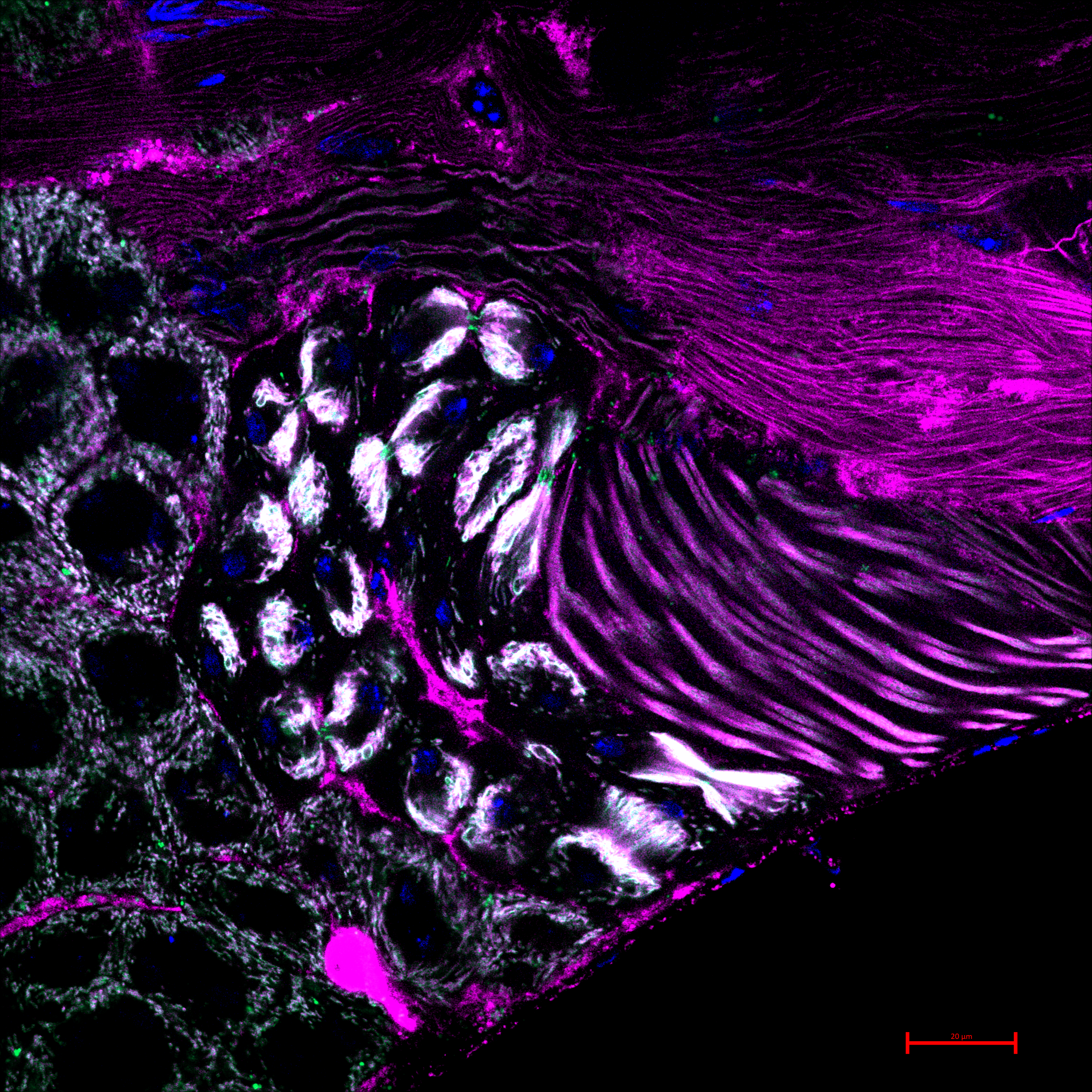

Supplement: Supplementary file 5 — Source data Fig. 3 [file 44318_2025_378_MOESM5_ESM.zip › Figure 3/3C/PB-mCherry tom20 GFP 4.tif]

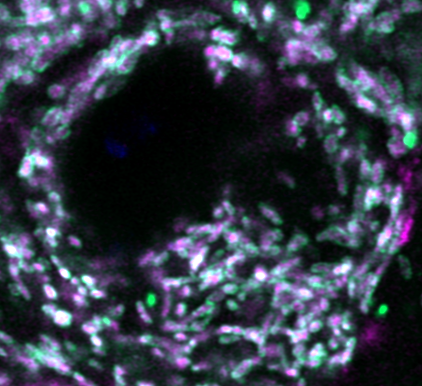

Supplement: Supplementary file 5 — Source data Fig. 3 [file 44318_2025_378_MOESM5_ESM.zip › Figure 3/3D/Tom20 and PB-mCherry.tif]

Fig 3A

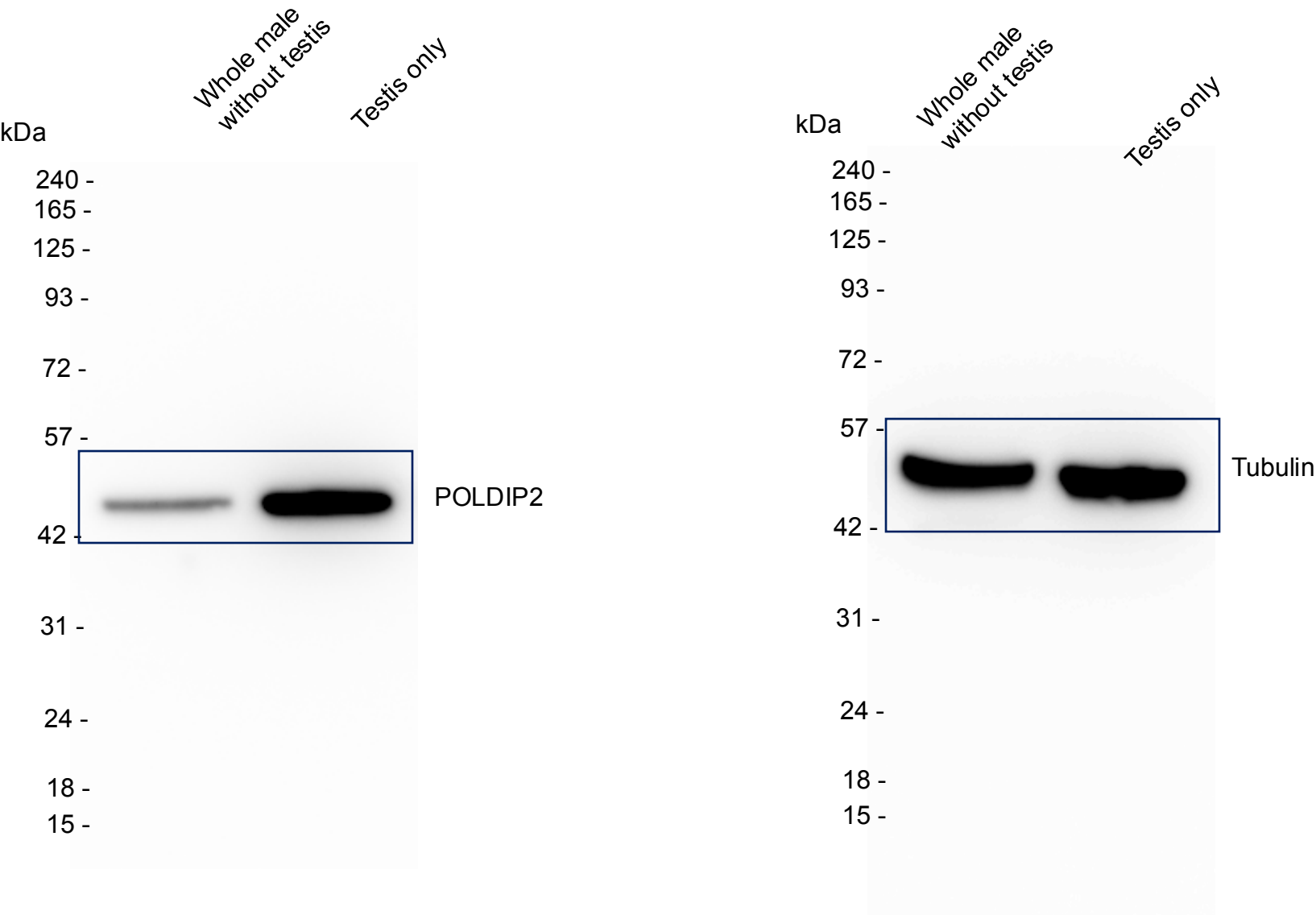

Supplement: Supplementary file 5 — Source data Fig. 3 [file 44318_2025_378_MOESM5_ESM.zip › Figure 3/3A/POLDIP2 levels in testis and soma.pdf]

Fig 3F

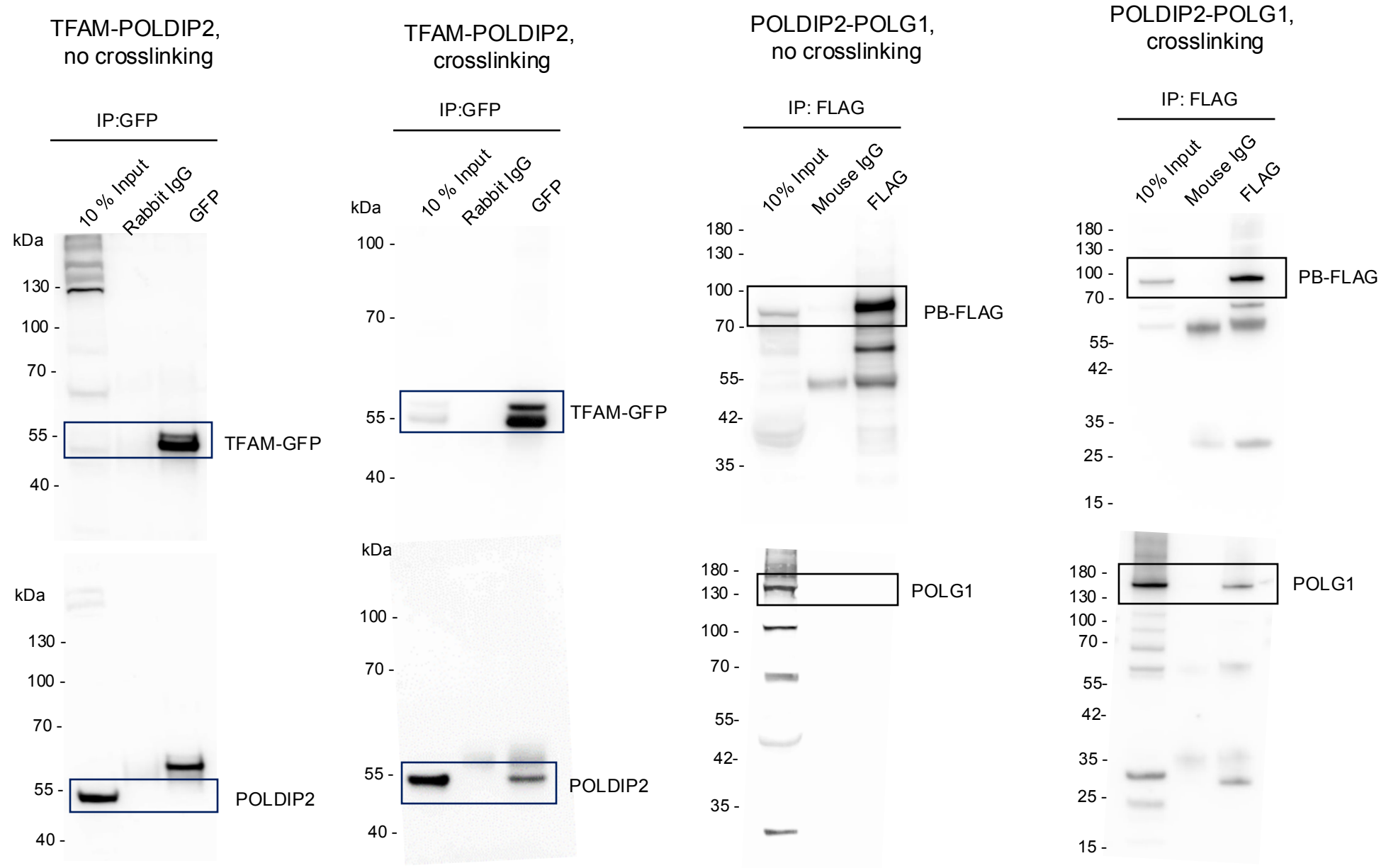

Supplement: Supplementary file 5 — Source data Fig. 3 [file 44318_2025_378_MOESM5_ESM.zip › Figure 3/3F/co-IP to probe interaction between POLDIP2 and TFAM or POLG1.pdf]

Fig 4B

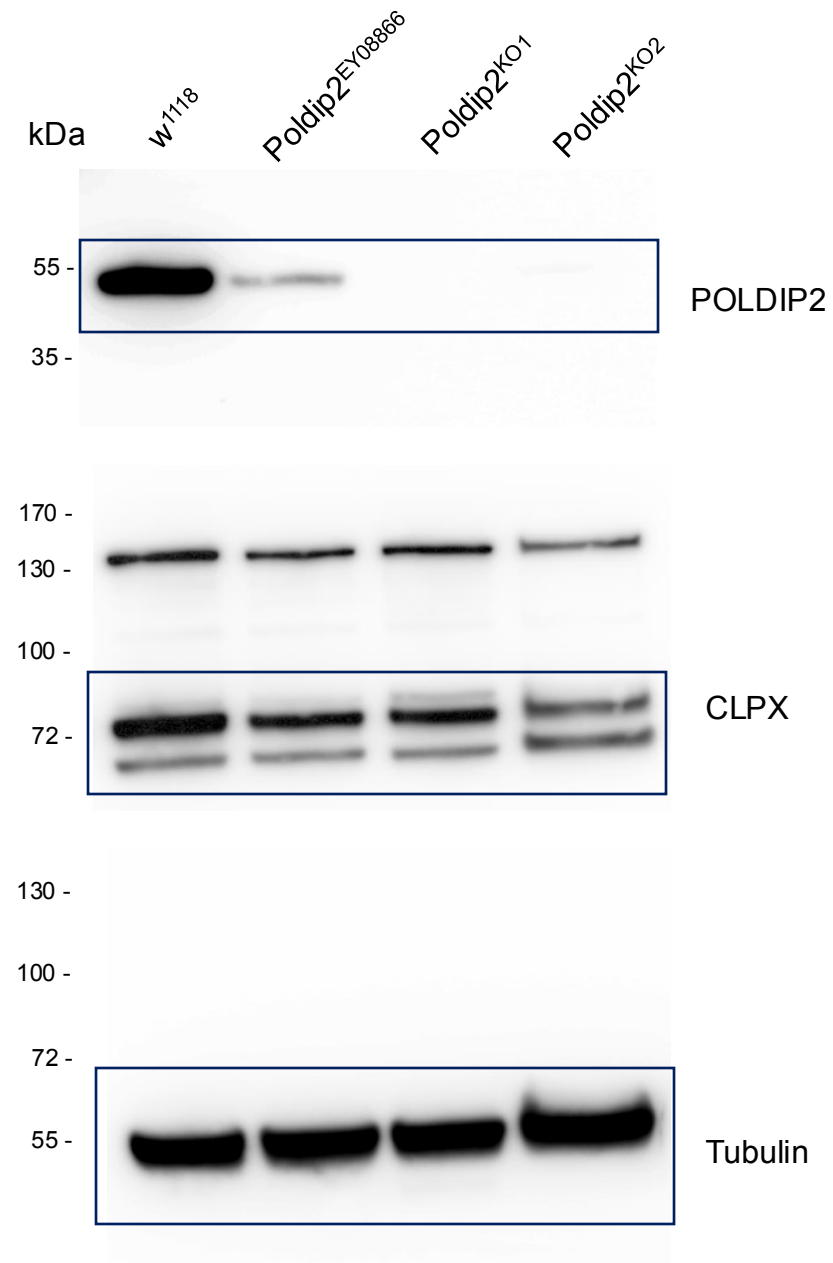

Supplement: Supplementary file 6 — Source data Fig. 4 [file 44318_2025_378_MOESM6_ESM.zip › Figure 4/4B/CLPX levels in POLDIP2 mutants.pdf]

Fig 4C

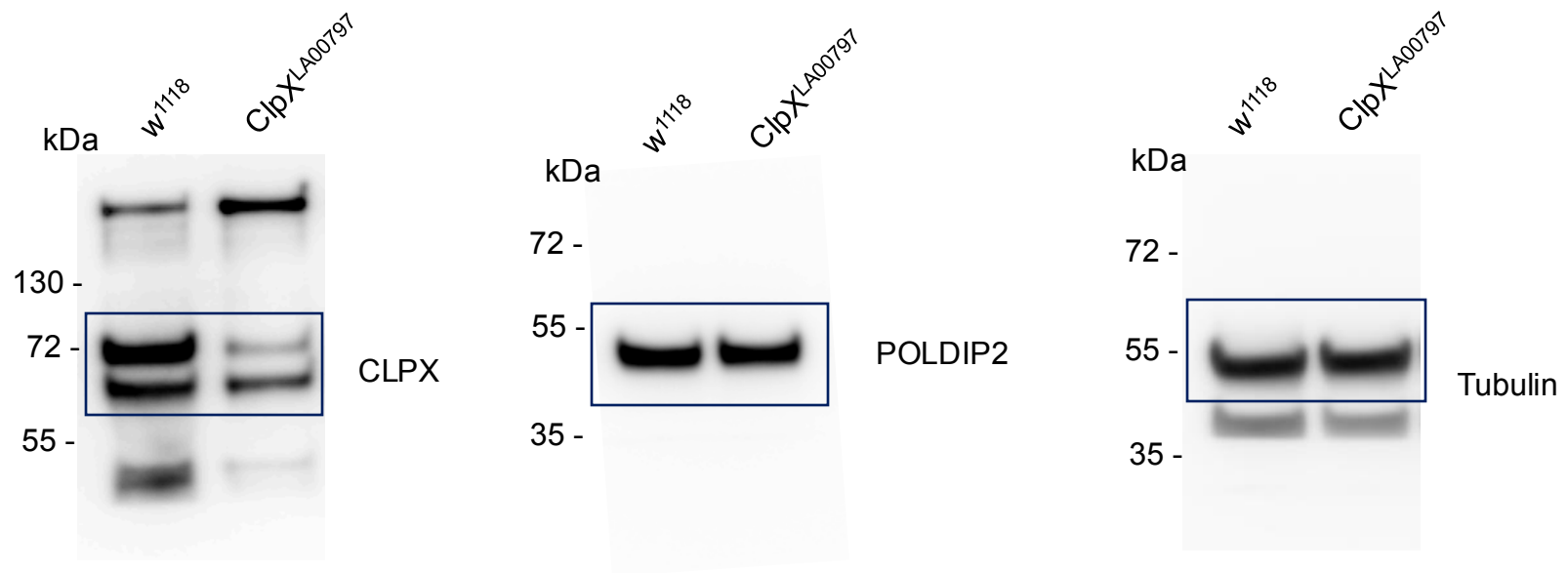

Supplement: Supplementary file 6 — Source data Fig. 4 [file 44318_2025_378_MOESM6_ESM.zip › Figure 4/4C/CLPX and POLDIP2 levels in ClpX mutant.pdf]

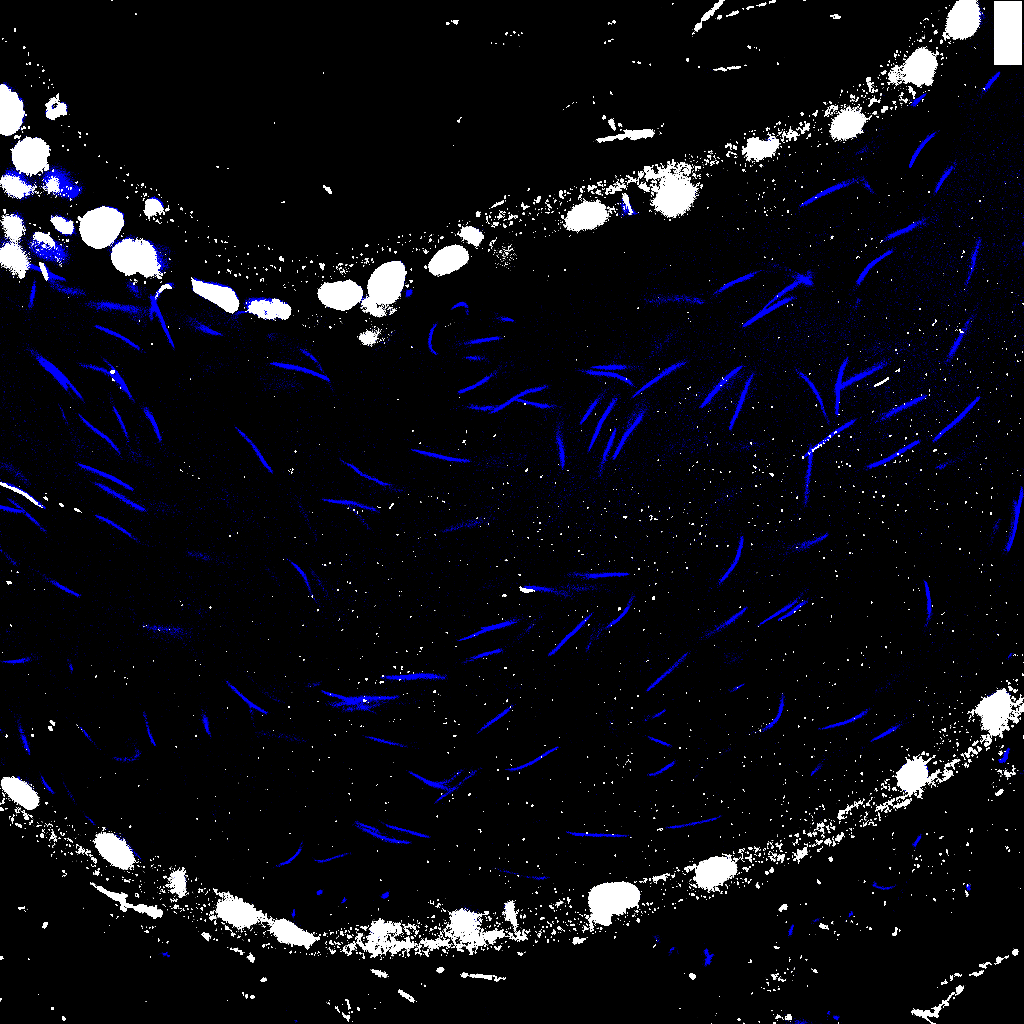

Supplement: Supplementary file 6 — Source data Fig. 4 [file 44318_2025_378_MOESM6_ESM.zip › Figure 4/4D/ClpX mutant dsDNA & DAPI.tif]

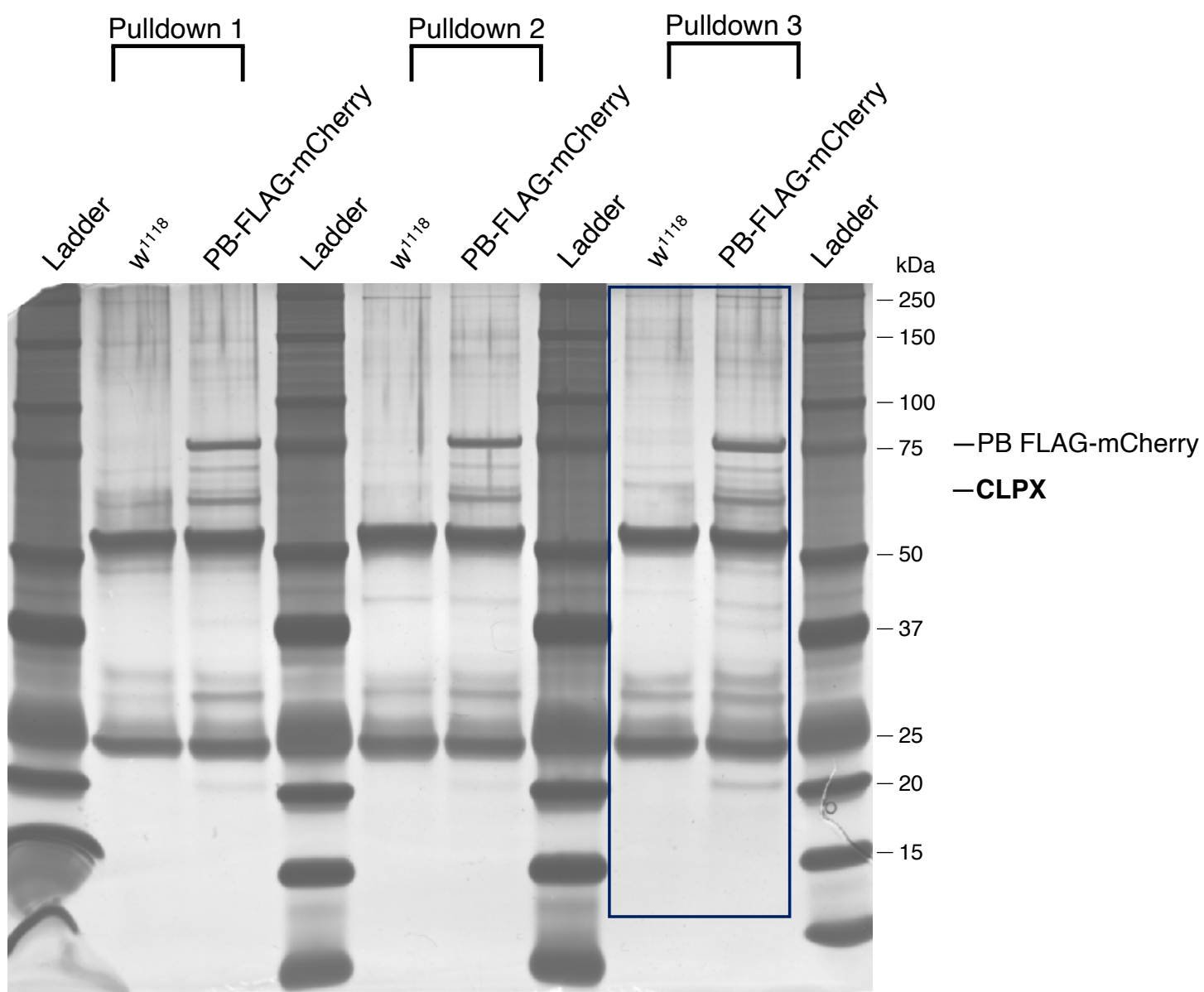

Supplement: Supplementary file 6 — Source data Fig. 4 [file 44318_2025_378_MOESM6_ESM.zip › Figure 4/4A/Pull down three replicas.pdf]

Fig 4F

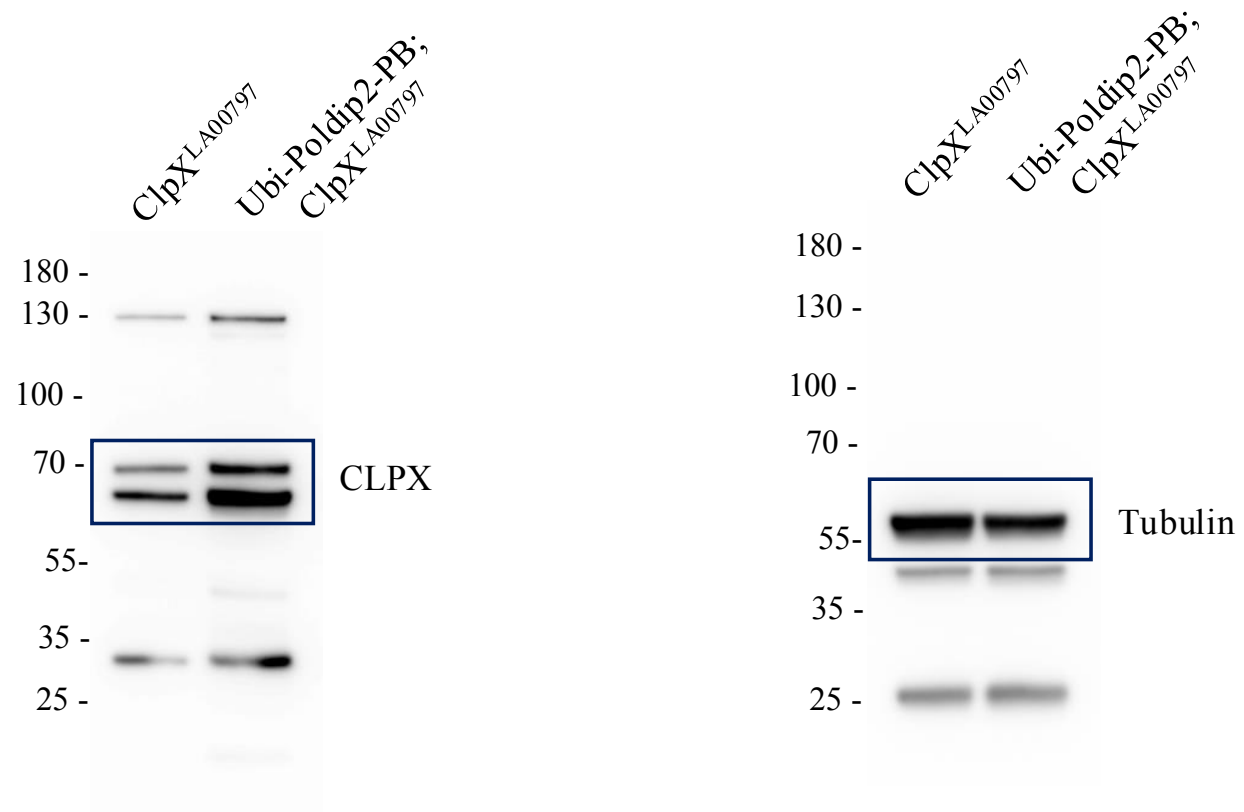

Supplement: Supplementary file 6 — Source data Fig. 4 [file 44318_2025_378_MOESM6_ESM.zip › Figure 4/4F/CLPX level in Ubi-POLDIP2.pdf]

Fig 4H

Endogenous TFAM & POLG1

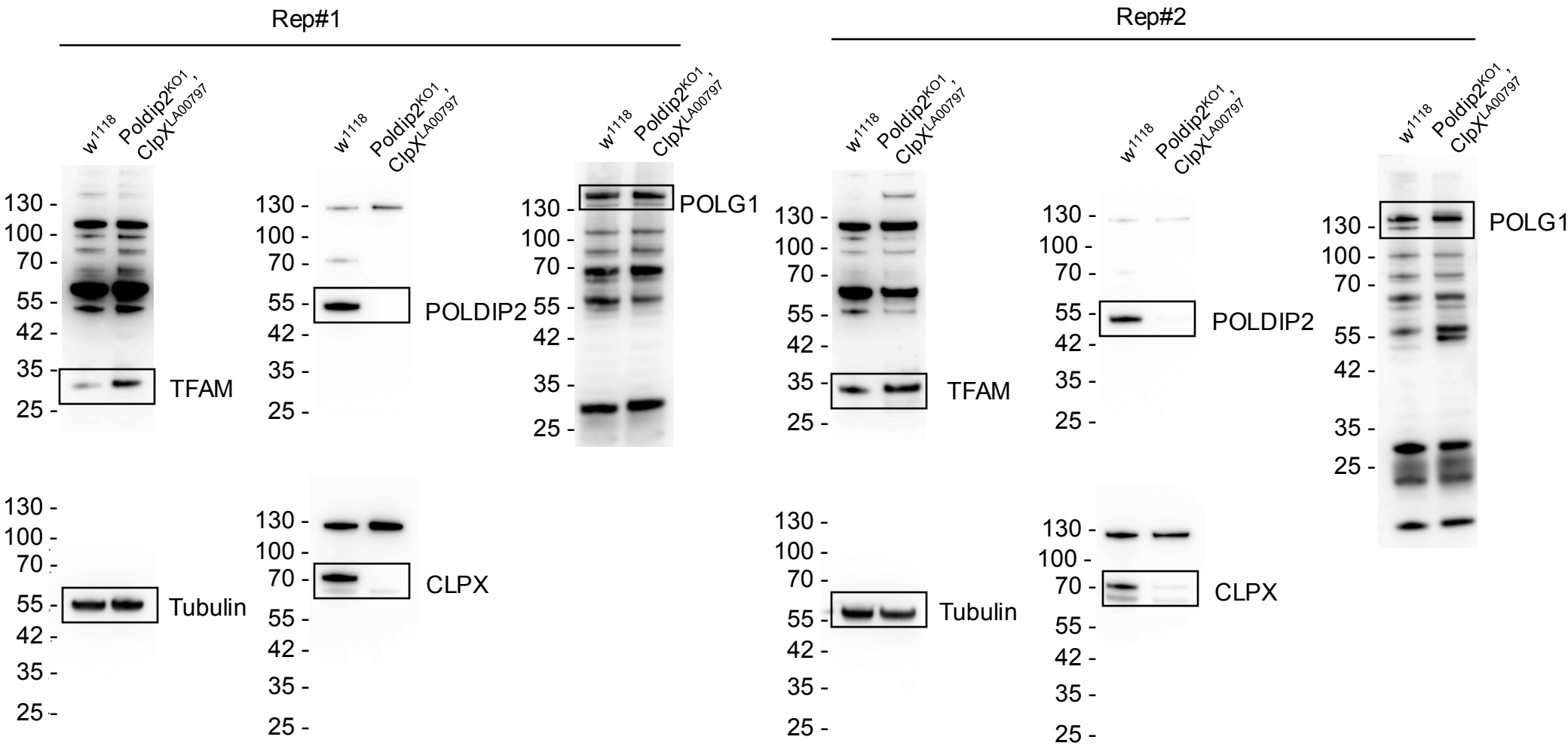

Supplement: Supplementary file 6 — Source data Fig. 4 [file 44318_2025_378_MOESM6_ESM.zip › Figure 4/4H/TFAM and POLG1 levels in the Poldip2 and ClpX double mutant.pdf]

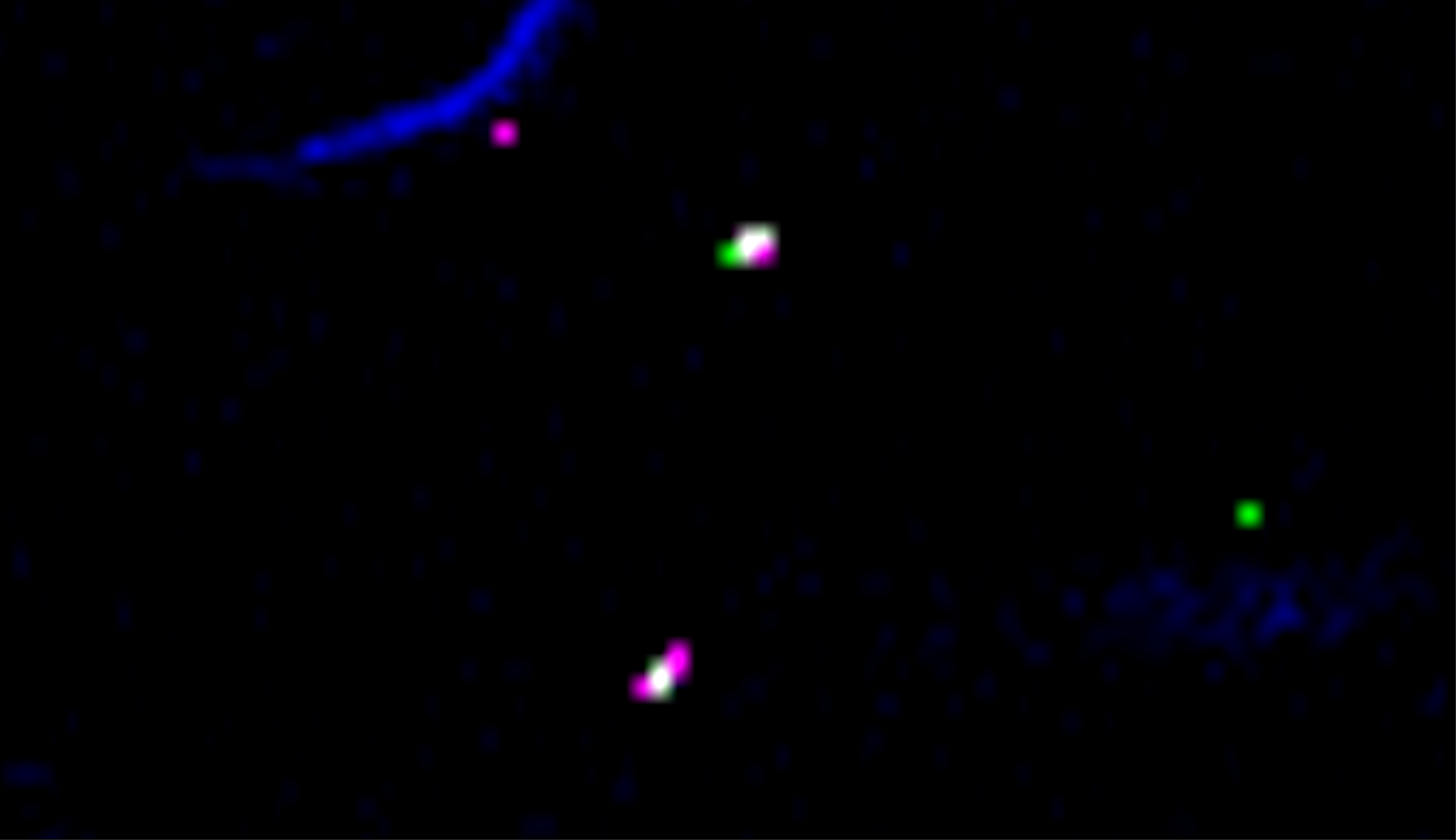

Supplement: Supplementary file 6 — Source data Fig. 4 [file 44318_2025_378_MOESM6_ESM.zip › Figure 4/4I/POLDIP2 KO1 tfam dsDNA_1.tif]

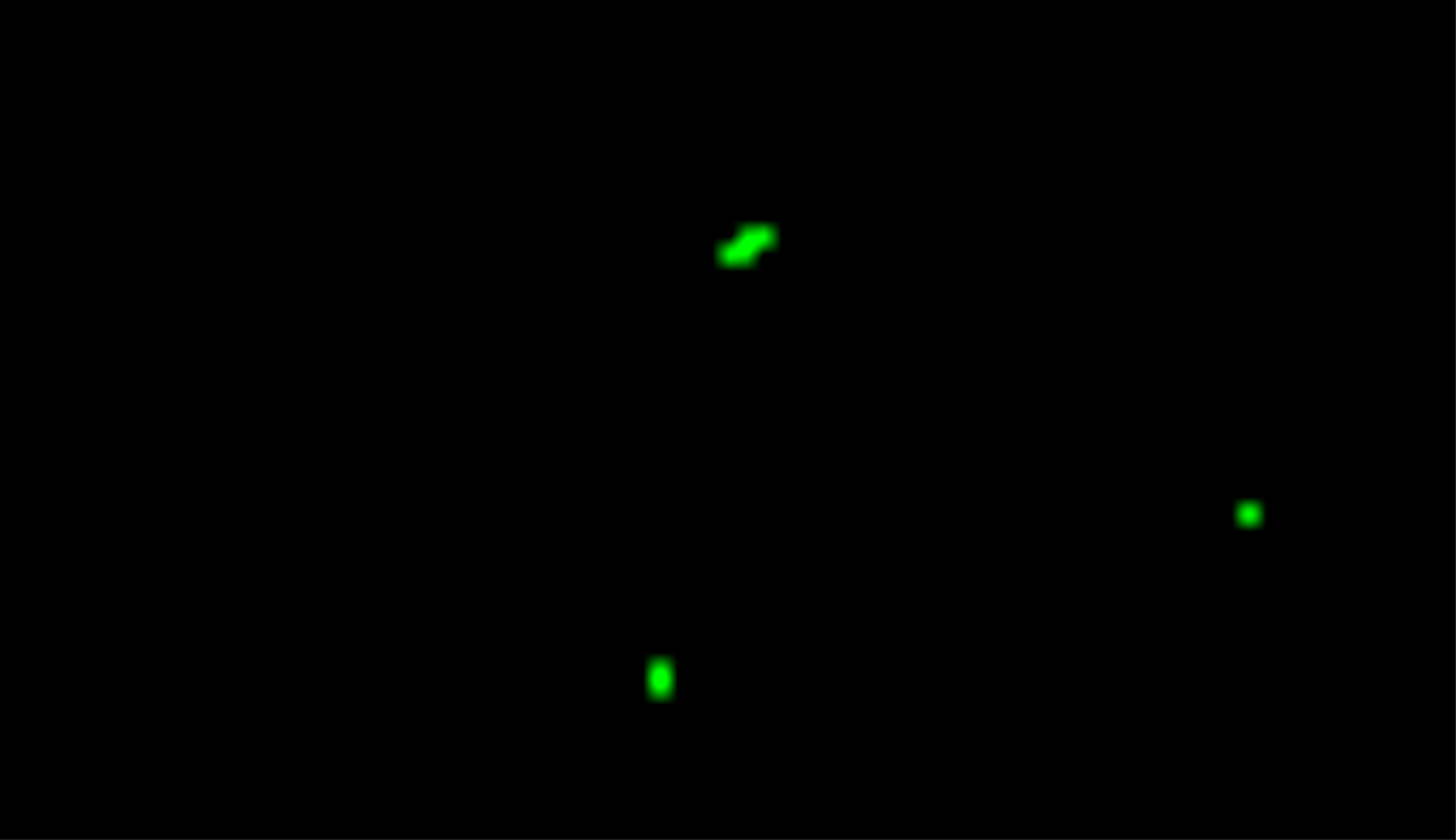

Supplement: Supplementary file 6 — Source data Fig. 4 [file 44318_2025_378_MOESM6_ESM.zip › Figure 4/4I/POLDIP2 KO1 tfam dsDNA_3.tif]

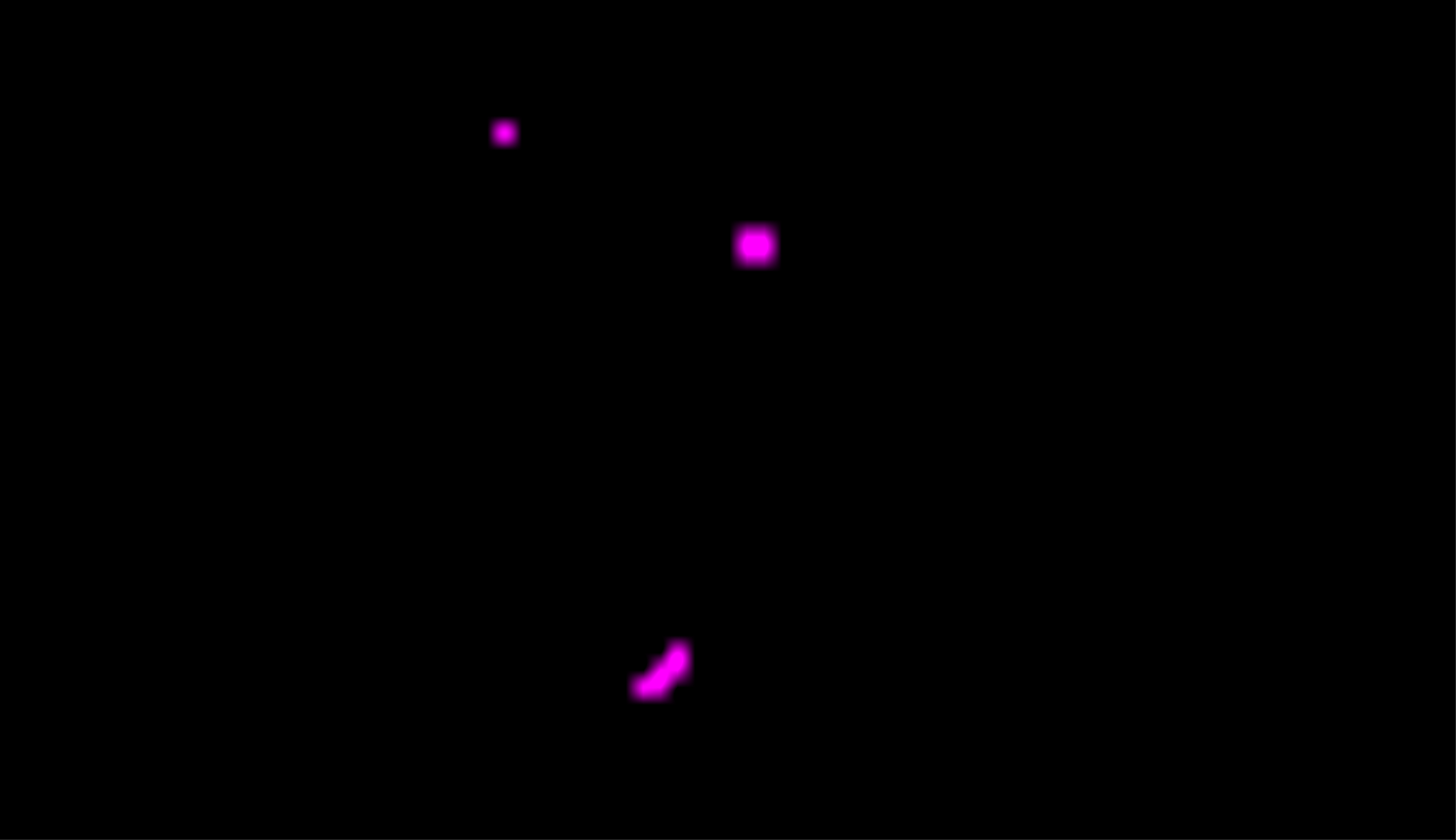

Supplement: Supplementary file 6 — Source data Fig. 4 [file 44318_2025_378_MOESM6_ESM.zip › Figure 4/4I/POLDIP2 KO1 tfam dsDNA_2.tif]
