# Supplementary material for: Poldip2 promotes mtDNA elimination during Drosophila spermatogenesis to ensure maternal inheritance
Source: EMBO J. 2025 Feb 11;44(6):1724–48. doi: 10.1038/s44318-025-00378-4 (PMC11914606; doi:10.1038/s44318-025-00378-4)
Supplement: Supplementary file 5 — Source data Fig. 3 [file 44318_2025_378_MOESM5_ESM.zip › Figure 3/3E/Poldip2 PK assay fly WB.pdf]

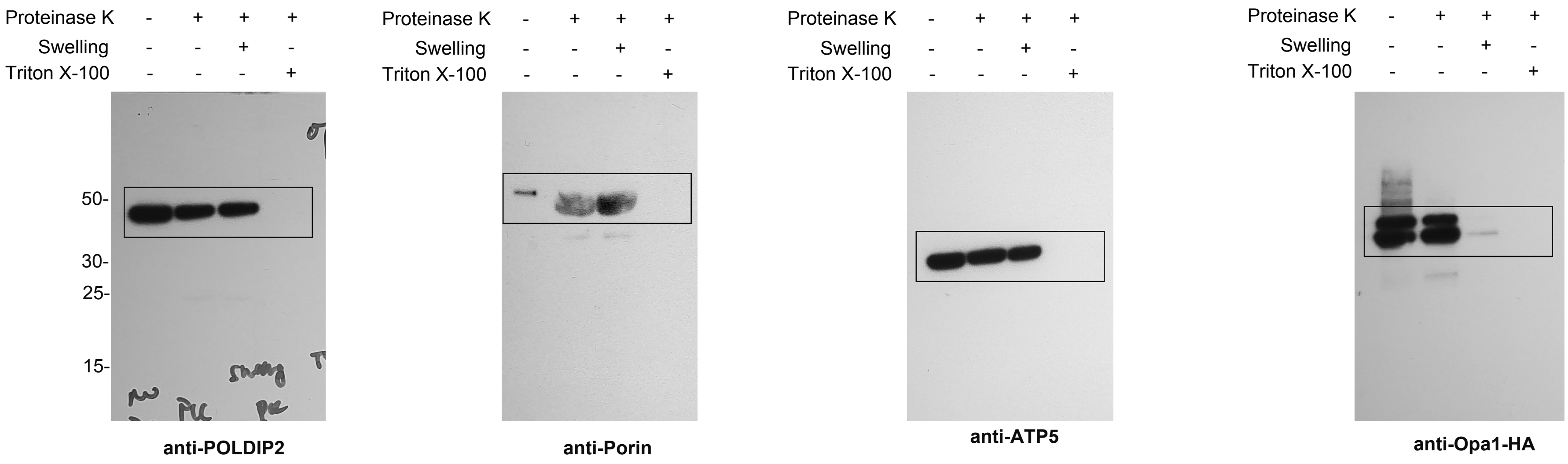
Proteinase K - + + +
Swelling - - + -
Triton X-100 - - - +
50-
30-
25-
15-
anti-POLDIP2
Proteinase K - + + +
Swelling - - + -
Triton X-100 - - - +
anti-Porin
Proteinase K - + + +
Swelling - - + -
Triton X-100 - - - +
anti-ATP5
Proteinase K - + + +
Swelling - - + -
Triton X-100 - - - +
anti-Opa1-HA
