# Supplementary material for: Poldip2 promotes mtDNA elimination during Drosophila spermatogenesis to ensure maternal inheritance
Source: EMBO J. 2025 Feb 11;44(6):1724–48. doi: 10.1038/s44318-025-00378-4 (PMC11914606; doi:10.1038/s44318-025-00378-4)
Supplement: Supplementary file 7 — Expanded View Figures [file 44318_2025_378_MOESM7_ESM.pdf]

Expanded View Figures

Figure EV1. The EMS screen used to identify mutant lines retaining paternal mtDNA in mature sperm.

(**A**) The cross scheme for establishing ~10,000 EMS lines carrying random mutations on the 2nd or 3rd chromosome of *D. melanogaster*. The following genotypes were used as the parental lines for the EMS mutagenesis: 1) *FRT40A* (Chr. 2L); *sqh-mitoYFP*, 2) *FRTG13* (Chr. 2R); *sqh-mitoYFP*, 3) *ubi-mtSSB-GFP*; *FRT2A* (Chr. 3L), and 4) *ubi-mtSSB-RFP*; *FRT82B* (Chr. 3R). Fly lines with balancer chromosomes used for subsequent crosses to establish individual EMS lines are as follows: 1) *Kr*^{If}*/CyO*; *sqh-mitoYFP* (Chr. 2L and Chr. 2R), 2) *ubi-mtSSB-GFP*; *MKRS/TM6B* (Chr. 3L), and 3) *ubi-mtSSB-RFP*; *MKRS/TM6B* (Chr. 3R). (**B**) Representative images showing DAPI, dsDNA, mtSSB-GFP and PicoGreen signals in spermatocytes and spermatids. Scale bars: 10 µm.

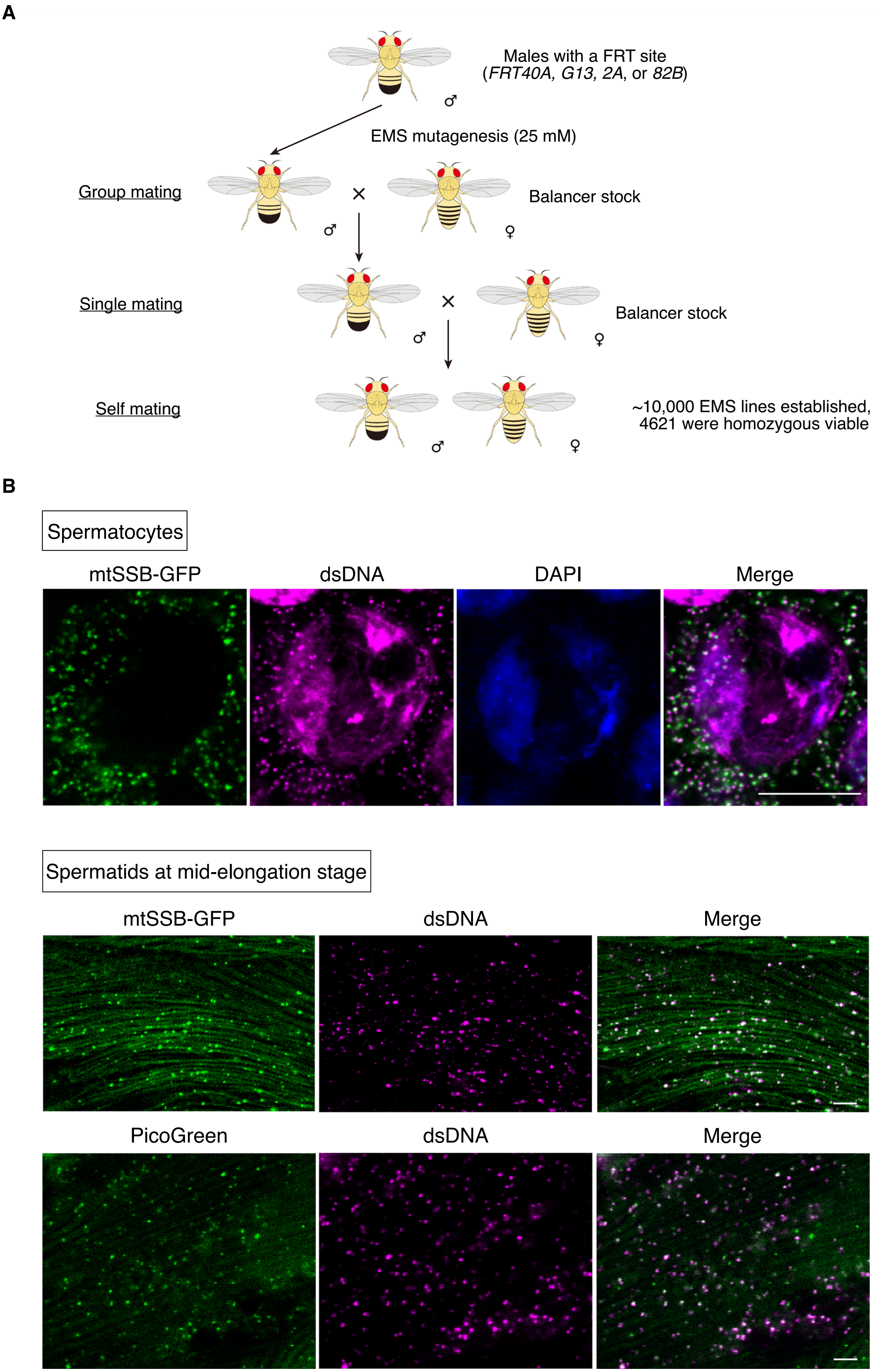
A
Males with a FRT site
(FRT40A, G13, 2A, or 82B)
♂
EMS mutagenesis (25 mM)
Group mating
×
Balancer stock
♂
♀
Single mating
×
Balancer stock
♂
♀
Self mating
~10,000 EMS lines established,
4621 were homozygous viable
♂
♀
B
Spermatocytes
mtSSB-GFP
dsDNA
DAPI
Merge
Spermatids at mid-elongation stage
mtSSB-GFP
dsDNA
Merge
PicoGreen
dsDNA
Merge

 © The Author(s)

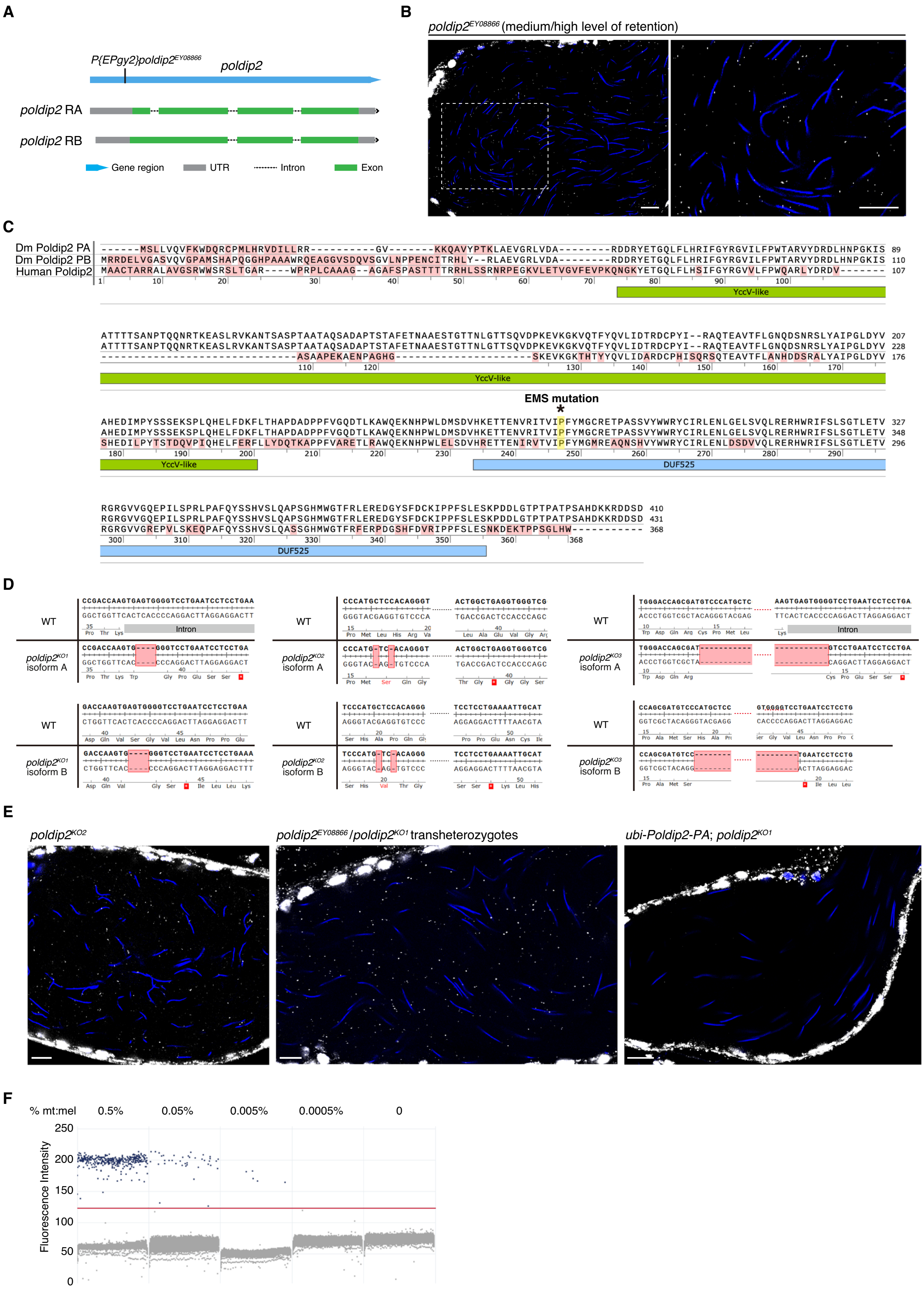
A
P{EPgy2}poldip2EY08866
poldip2
poldip2 RA
poldip2 RB
Gene region
UTR
Intron
Exon
B
poldip2EY08866 (medium/high level of retention)
C
Dm Poldip2 PA
Dm Poldip2 PB
Human Poldip2
YccV-like
EMS mutation
DUF525
D
WT
poldip2KO1 isoform A
poldip2KO2 isoform A
poldip2KO3 isoform A
poldip2KO1 isoform B
poldip2KO2 isoform B
poldip2KO3 isoform B
Intron
E
poldip2KO2
poldip2EY08866/poldip2KO1 transheterozygotes
ubi-Poldip2-PA; poldip2KO1
F
% mt:mel
0.5%
0.05%
0.005%
0.0005%
0
Fluorescence Intensity
250
200
150
100
50
0

Figure EV2. Poldip2 is required for mtDNA removal in *Drosophila* spermatids.

(**A**) Schematic of the P-element insertion site for the *poldip2*^{EY08866} line. (**B**) A representative image showing the level of mtDNA retention in seminal vesicles of *poldip2*^{EY08866} mutant. The samples were stained with anti-dsDNA antibodies (white, mtDNA) and DAPI (blue, nuclear DNA). Scale bars: 10 µm. (**C**) The alignment of the Poldip2 sequence of two fly isoforms and the human protein, with the positions of YccV-like and DUF525 domains annotated. The *EMS-23* carries a point mutation that converts the highly conserved proline to serine in the DUF525 domain (highlighted in yellow with an asterisk mark). (**D**) Sequences of *poldip2* mutants generated in this study. (**E**) Representative images with the level of mtDNA retention in seminal vesicles of *poldip*^{KO2}, *poldip2*^{EY08866}/*poldip2*^{KO1} transheterozygotes, and *ubi-Poldip2-PA-FLAG-mCherry*; *poldip2*^{KO1} flies. The samples were stained with anti-dsDNA antibodies (white, mtDNA) and DAPI (blue, nuclear DNA). Scale bars: 10 µm. (**F**) Plots illustrating the detection power of our ddPCR assay. *D. melanogaster* mtDNA was mixed with *D. yakuba* mtDNA to generate samples with 0.5%, 0.05%, 0.005% and 0.0005% of mt:mel before the run. The red line marks the threshold, above which droplets were considered positive for mt:mel (i.e. paternal mtDNA).

 © The Author(s)

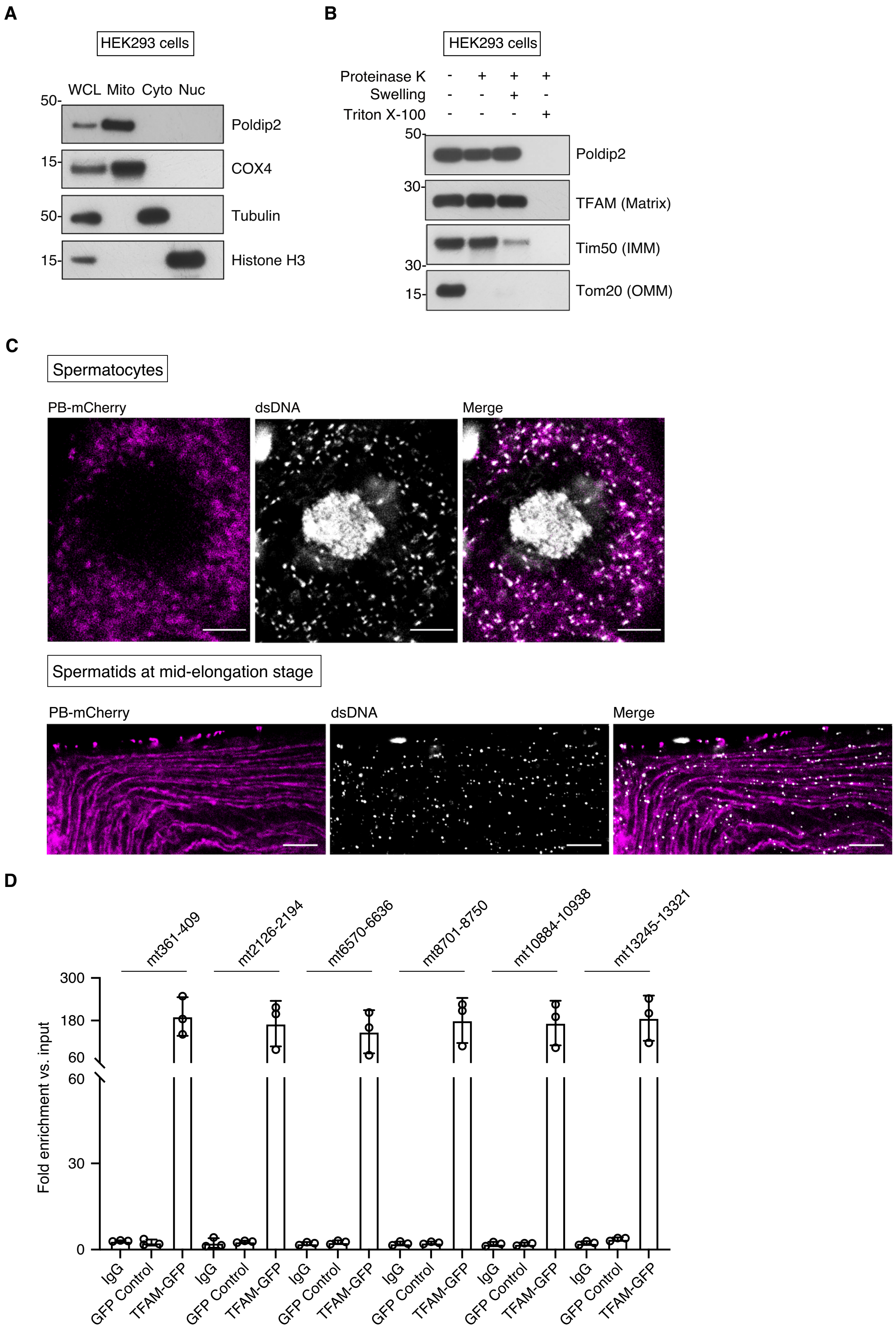
A
HEK293 cells
WCL Mito Cyto Nuc
50-
Poldip2
15-
COX4
50-
Tubulin
15-
Histone H3
B
HEK293 cells
Proteinase K - + + +
Swelling - - + -
Triton X-100 - - - +
50-
Poldip2
30-
TFAM (Matrix)
Tim50 (IMM)
30-
15-
Tom20 (OMM)
C
Spermatocytes
PB-mCherry
dsDNA
Merge
Spermatids at mid-elongation stage
PB-mCherry
dsDNA
Merge
D
mt361-409
mt2126-2194
mt6570-6636
mt8701-8750
mt10884-10938
mt13245-13321
Fold enrichment vs. input
300
180
60
60
30
0
IgG
GFP Control
TFAM-GFP

Figure EV3. Poldip2 is a mitochondrial matrix protein.

(**A**) Immunoblots of whole-cell lysate (WCL), mitochondrial (Mito, anti-COX4), cytoplasmic (Cyto, anti-Tubulin) and nuclear (Nuc, anti-Histone H3) fractions of HEK293 cells to reveal the subcellar enrichment of Poldip2. (**B**) Immunoblots of proteinase K protection assay in HEK293 cells. TFAM, Tim50, and Tom20 were blotted as the mitochondrial matrix, inner membrane (IMM) and outer membrane (OMM) marker, respectively. (**C**) Confocal images showing Poldip2-mCherry signals (magenta) and dsDNA foci (white) in spermatocytes and spermatids of *ubi-Poldip2-PB-FLAG-mCherry* flies. Scale bars: 5 µm. (**D**) ChIP-qPCR measuring the mtDNA enrichment levels with TFAM-GFP immunoprecipitation in flies ($n = 3$ biological replicates). Embryos were used to obtain sufficient materials. Data were normalised to input DNA. IgG control: immunoprecipitating TFAM-GFP samples with IgG; GFP control: immunoprecipitating wild-type samples (i.e. no GFP expression) with anti-GFP antibodies to control for unspecific bindings between anti-GFP antibodies and mtDNA during the assay; TFAM GFP: immunoprecipitating TFAM with GFP antibodies. Three independent ChIP assays were performed for TFAM-GFP, and the mtDNA enrichment level was measured by qPCR using six pairs of primers binding to different regions of mtDNA. Data: mean ± SD.

 © The Author(s)

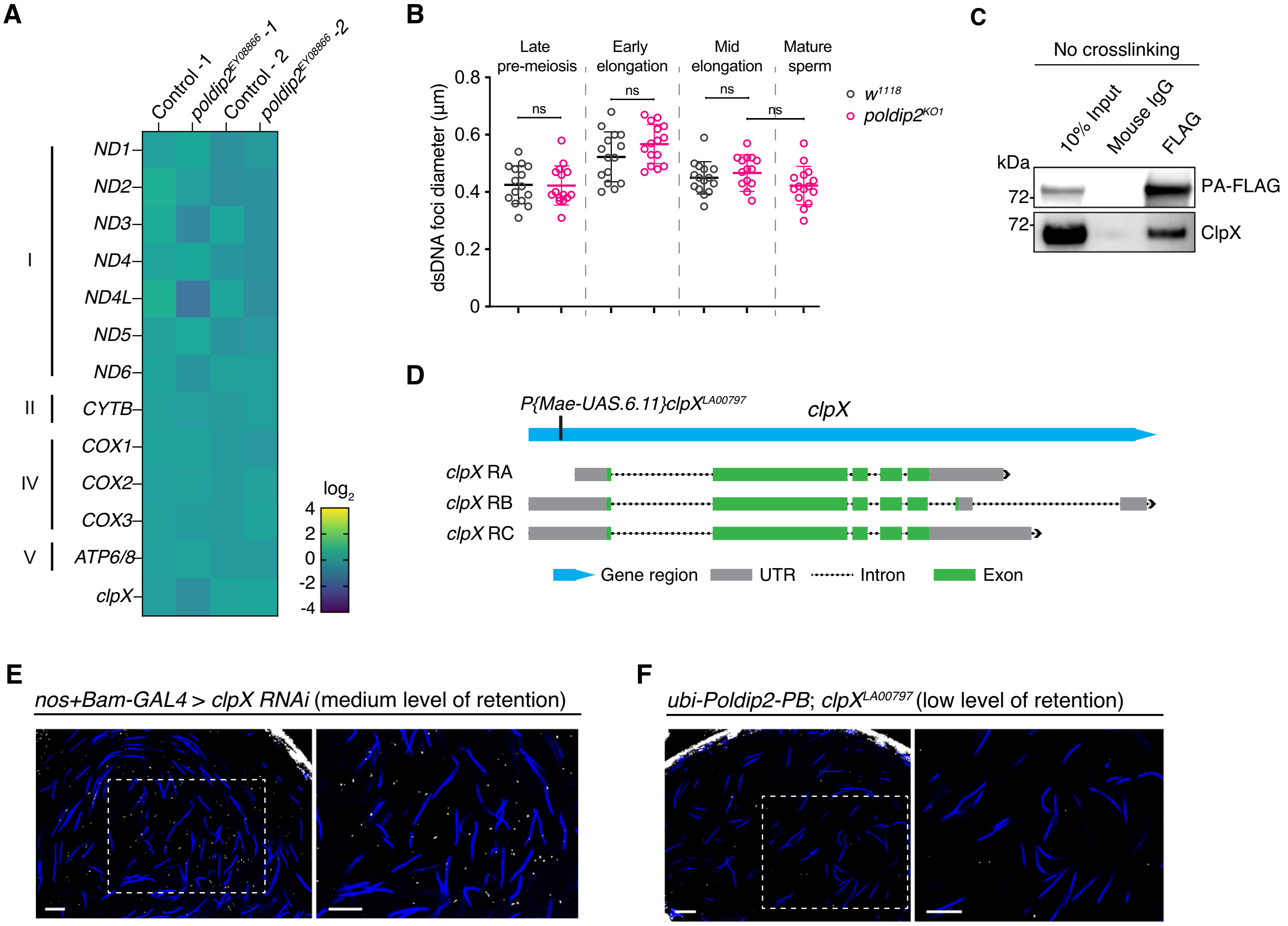

Figure EV4. ClpX and Poldip2 co-regulate mtDNA elimination in *Drosophila* spermatids.

(**A**) A heatmap with mRNA levels of mtDNA genes in control and *poldip2*^{EY08866} testes. (**B**) Diameters of mtDNA nucleoid at different stages of spermatogenesis ($n = 15$ biological replicates). Data: mean ± SD, unpaired Student's t-test, $P = 0.9145$, 0.1285, 0.4542 and 0.0795, respectively. (**C**) Immunoblots of co-IP using anti-FLAG antibodies. Testes isolated from *ubi-Poldip2-PA-FLAG-mCherry* flies were used, and ClpX was blotted. (**D**) Schematic of the P-element insertion site for the *clpX*^{LA00797} line. (**E**) A representative image of seminal vesicles isolated from *clpX* RNAi; *nos+Bam-GAL4* flies stained with anti-dsDNA antibodies (white) and DAPI (blue). Scale bars: 10 μm. (**F**) A representative image of seminal vesicles isolated from *ubi-Poldip2-PB*; *clpX*^{LA00797} flies stained with anti-dsDNA antibodies (white) and DAPI (blue). Scale bars: 10 μm.

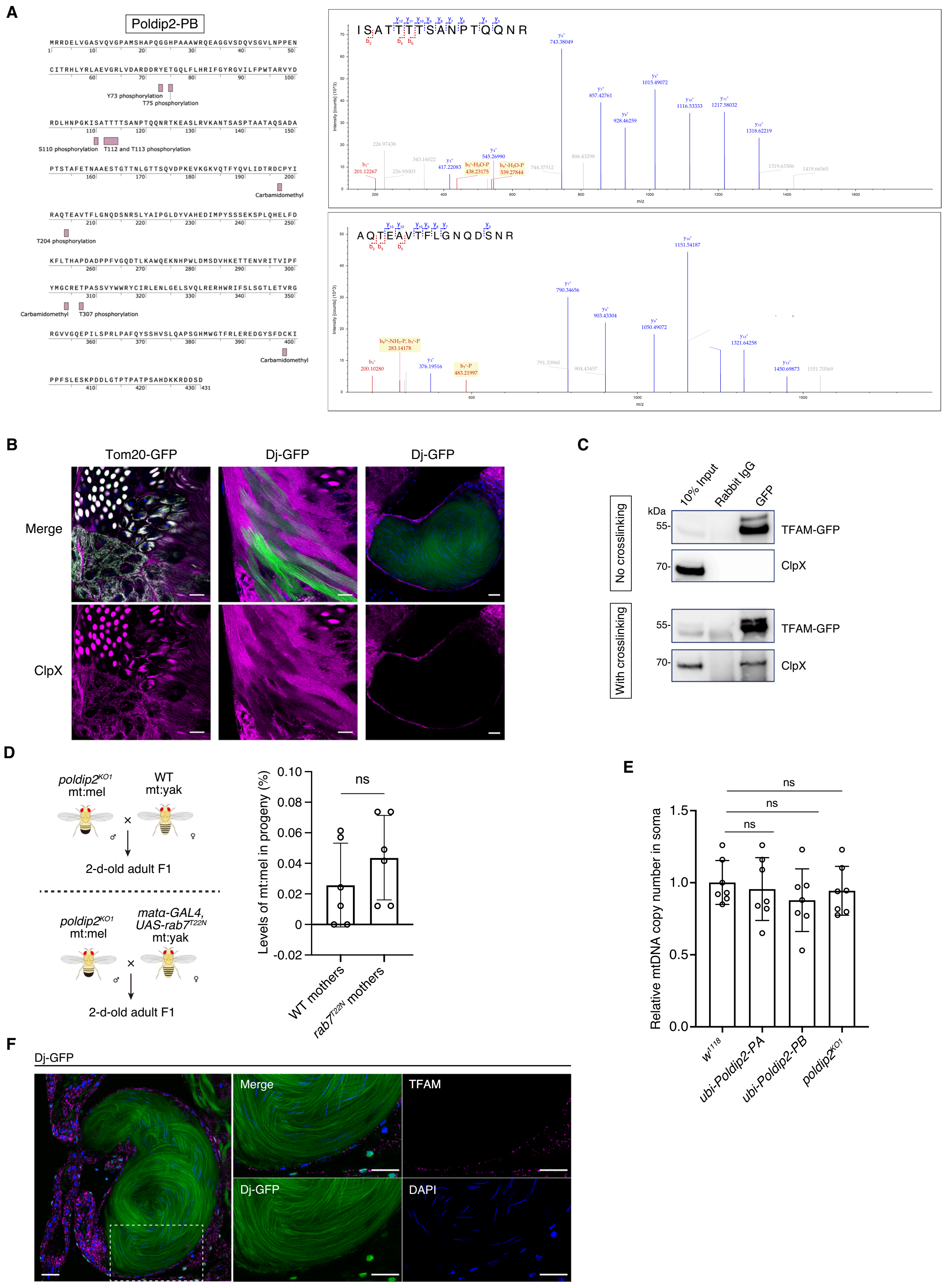
A
Poldip2-PB
Y73 phosphorylation
T75 phosphorylation
S110 phosphorylation
T112 and T113 phosphorylation
Carbamidomethyl
T204 phosphorylation
T307 phosphorylation
I S A T T T T S A N P T Q Q N R
A Q T E A V T F L G N Q D S N R
Intensity [counts] (10^3)
m/z
B
Tom20-GFP
Dj-GFP
Dj-GFP
Merge
ClpX
C
10% Input
Rabbit IgG
GFP
kDa
No crosslinking
With crosslinking
55-
70-
TFAM-GFP
ClpX
D
poldip2KO1
mt:mel
WT
mt:yak
2-d-old adult F1
mata-GAL4,
UAS-rab7T22N
mt:yak
Levels of mt:mel in progeny (%)
ns
WT mothers
rab7T22N mothers
E
Relative mtDNA copy number in soma
ns
w1118
ubi-Poldip2-PA
ubi-Poldip2-PB
poldip2KO1
F
Dj-GFP
Merge
TFAM
Dj-GFP
DAPI

Figure EV5. The role of Poldip2 and ClpX in regulating mtDNA dynamics during *Drosophila* spermatogenesis.

(**A**) Post-translational modification sites of Poldip2-PB identified by mass spectrometry analyses. The left panel notes all the phosphorylation and carbamidomethylation sites based on the MS1 spectra. The right panel is the two MS2 spectra plots for T112/113 and T204 phosphorylation sites. (**B**) Representative images showing ClpX expression in spermatocytes, spermatids and mature sperm. ClpX (magenta) was visualised by immunostaining, whereas *Dj-GFP* and *ubi-Tom20-GFP* (green) were used to visualise mitochondria. Samples were also stained with DAPI (blue). Scale bars: 20 µm. (**C**) Immunoblots of co-IP with anti-GFP antibodies to probe interactions between TFAM or ClpX in testes with or without cross-linking. (**D**) Percentages of paternal mtDNA in 2-d-old adult progeny of *poldip2*^{KO1} males when crossed to wild-type females or females expressing Rab7^{T22N} ($n = 6$ crosses). Data: mean ± SD, unpaired Student's t-test, $P = 0.2859$. (**E**) The relative mtDNA copy number in soma measured by qPCR for *w*^{1118}, *ubi-Poldip2-PA*, *ubi-Poldip2-PB* and *poldip2*^{KO1} males. The number was normalised to *w*^{1118} samples ($n = 7$). Data: mean ± SD, One-Way ANOVA, $P = 0.9438$, 0.4985 and 0.8983, respectively. (**F**) Representative images showing TFAM localisation in seminal vesicles isolated from *Dj-GFP* flies. TFAM (magenta) was visualised by immunostaining, whereas *Dj-GFP* (green) was used to visualise mitochondria. Samples were also stained with DAPI (blue). Scale bars: 20 µm.
